# Age prediction by deep learning applied to Greenland halibut (*Reinhardtius hippoglossoides*) otolith images

**DOI:** 10.1371/journal.pone.0277244

**Published:** 2022-11-04

**Authors:** Iver Martinsen, Alf Harbitz, Filippo Maria Bianchi

**Affiliations:** 1 Department of Mathematics and Statistics, University of Tromsø, Tromsø, Norway; 2 Department of Deep-Water Species and Cartilaginous Fish, Institute of Marine Research, Tromsø, Norway; 3 NORCE, The Norwegian Research Centre AS, Bergen, Norway; Sichuan University, CHINA

## Abstract

Otoliths (ear-stones) in the inner ears of vertebrates containing visible year zones are used extensively to determine fish age. Analysis of otoliths is a time-consuming and difficult task that requires the education of human experts. Human age estimates are inconsistent, as several readings by the same human expert might result in different ages assigned to the same otolith, in addition to an inherent bias between readers. To improve efficiency and resolve inconsistent results in the age reading from otolith images by human experts, an automated procedure based on convolutional neural networks (CNNs), a class of deep learning models suitable for image processing, is investigated. We applied CNNs that perform image regression to estimate the age of Greenland halibut (*Reinhardtius hippoglossoides*) with good results for individual ages as well as the overall age distribution, with an average CV of about 10% relative to the read ages by experts. In addition, the density distribution of predicted ages resembles the density distribution of the ground truth. By using k*l-fold cross-validation, we test all available samples, and we show that the results are rather sensitive to the choice of test set. Finally, we apply explanation techniques to analyze the decision process of deep learning models. In particular, we produce heatmaps indicating which input features that are the most important in the computation of predicted age.

## Introduction

A reliable estimate of the age distribution of a fish stock is important in stock assessment and stock development modeling. This concerns for example the detection of recruitment of young fish as well as to monitor time series of the age distribution of the fish and the “survival” of strong year-classes. A year class in this context is the part of the stock “born” at a certain year.

**Fig 1:**
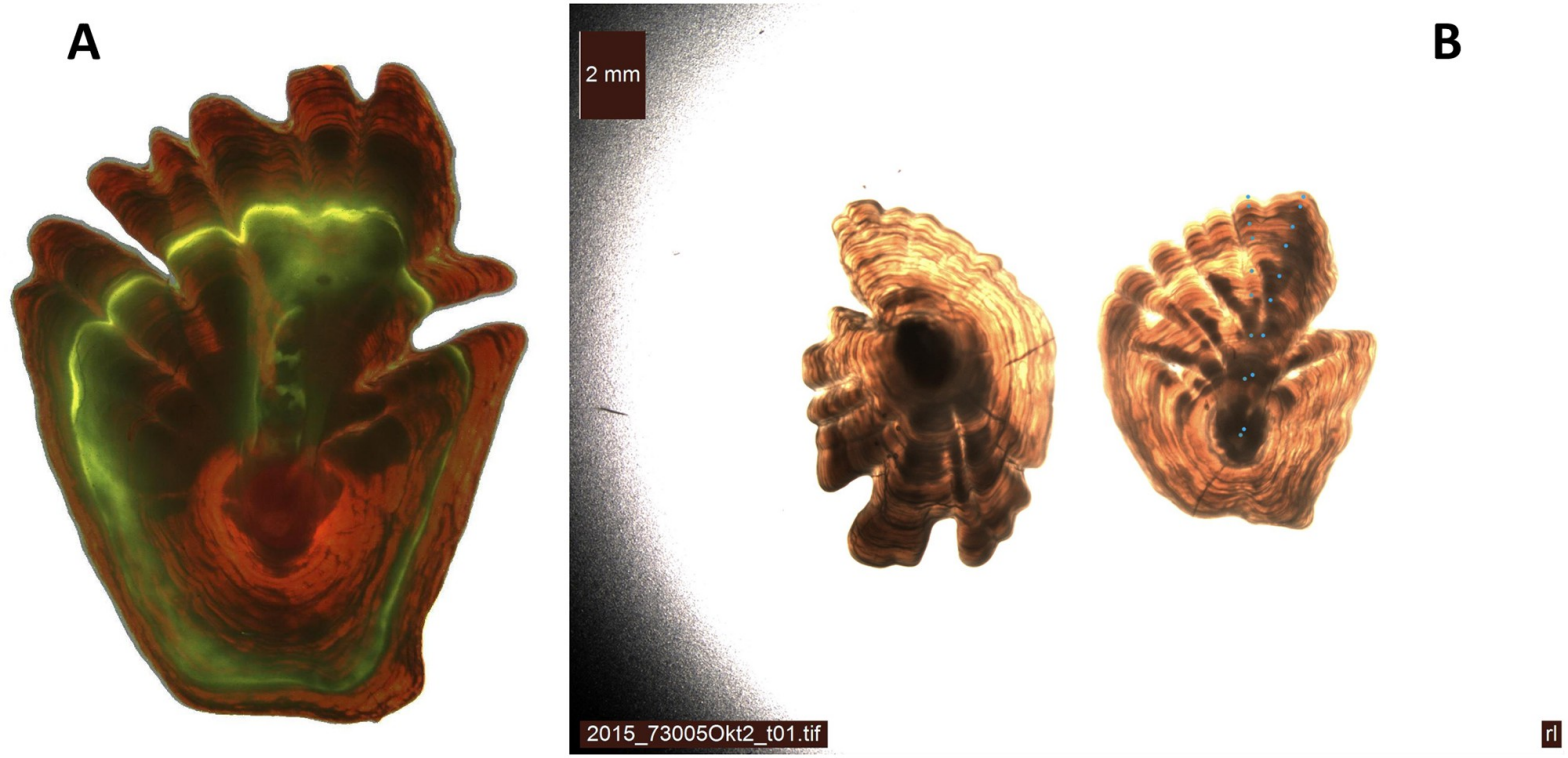
Greenland halibut otoliths. A: An example of a Greenland halibut otolith, where the otolith growth is revealed when recatching a fish that has previously been injected with OTC. B: Otoliths develop annual zones as the fish grows older. Back-light through the translucent Greenland halibut otolith reveals the otolith annual zones, which are counted to determine the age from the right otolith (two “parallel” dot series by two different readers).

For most species of fish, the most reliable method to estimate age is to count annual zones in otoliths, a three-dimensional calcified structure present in the inner ear of vertebrates [1]. More precisely, there are three pairs of otoliths, where the largest pair, *sagitta*, is used for age reading. This is done directly by microscope or by analyzing images of the otolith, either of the entire (thin) otolith or a cross-section through the core (“thick” otolith). Because the true age of wild fish is rarely known, a complicated challenge is to validate the reading method. A method applied to Greenland halibut (*Reinhardtius hippoglossoides*) is to inject fish with oxytetracycline (OTC). The OTC forms chemical bonds to calcified structures like otoliths and emit fluorescence under ultraviolet light [2], a fluorescent substance that settle to the otolith before the fish is released (Fig 1A). Then, since the time span between injection and recatch is known, one can validate the estimated age difference between injected age and recaught age [3].

Greenland halibut otolith images analyzed by the Norwegian Institute of Marine Research are examined by translucent light through the whole otolith in a direction along the upward (longest) extension from the core to the perimeter of the right sagitta otolith. In Fig 1B the age zone identifications of two different readers are shown by small dots.

Analysis of otoliths is performed by trained human experts, a process that is difficult and time consuming. The precision and bias of the age estimates vary between readers, and sometimes the same reader can estimate different ages for the same otolith when observing it at different times. Clearly, this often leads to inconsistent estimates. About 1 million otoliths are read each year around the world [4] and, thus, developing consistent and efficient age estimation methods is very important.

Artificial neural networks, which are effective in modeling complex relationships between high-dimensional inputs and labels in classification and regression problems, can be used for automatic age prediction. Among the different types of neural networks, CNNs are deep learning models suitable to process image data. The automatic analysis of otolith images using CNNs is consistent, in the sense that a trained deep learning model would produce the same output for the same input. This is a major advantage in comparison to estimates from human readers. A general review of the developments in deep learning and the adaptation of this technology to marine sciences is given in a recent survey [5]. The feasibility of using CNNs for automatic age estimation of fish otoliths has been confirmed by the results obtained for several different species. In particular, good results have been achieved for New Zealand fish species snapper and hoki [6] and the Mediterranean red mullet [7]. The latter study obtained excellent results using multitask learning, where length was used as the target variable for an auxiliary task, which is solved by the neural network in parallel to age estimation in order to avoid overfitting. The age of Greenland halibut otoliths has also been estimated with good results in recent studies [8–10]. For the Greenland halibut, a recent paper successfully demonstrated how one can predict fish age across otolith image labs based on a trained network at one lab by use of Domain Adaptation [10].

Explanation techniques allow us to verify the validity of a deep learning model and to interpret its decision process. For input images, this is often done by attributing relevance scores to the pixels of the input image, thereby creating a visualization of the pixels that mostly explain the predicted age. Marine biologists are particularly interested in understanding what features the model finds important rather than simply using the deep learning model as a black box. An example of explaining the decisions of deep neural networks used to predict the age of fish is given by [9], which reports heatmaps of the input images showing the relevance of each pixel.

The main objective of this work is to analyze the performance of a CNN in predicting fish ages from 4,243 Greenland halibut images provided by the Institute of Marine Research with read ages (labels) provided by experts. Although deep learning has been previously applied to estimate the age of Greenland halibuts [8, 9], the results reported so far were based on relatively small test sets that were arbitrarily chosen. To improve the robustness in the results, we perform an exhaustive cross-validation that utilizes all images for testing. By doing so, we are able to compute an unbiased estimate of the model performance (i.e., the average performance over all test sets) and asses to what extent the model performance depends on the choice of test data (i.e., the variability of performance across test sets). As a second contribution, we show that the age estimation can be improved by using sex as an additional input feature. Finally, we apply several explanation techniques to create heatmaps that highlight which input features are considered important in the decision-making for the model.

## Materials and methods

### Greenland halibut otolith data

**Fig 2:**
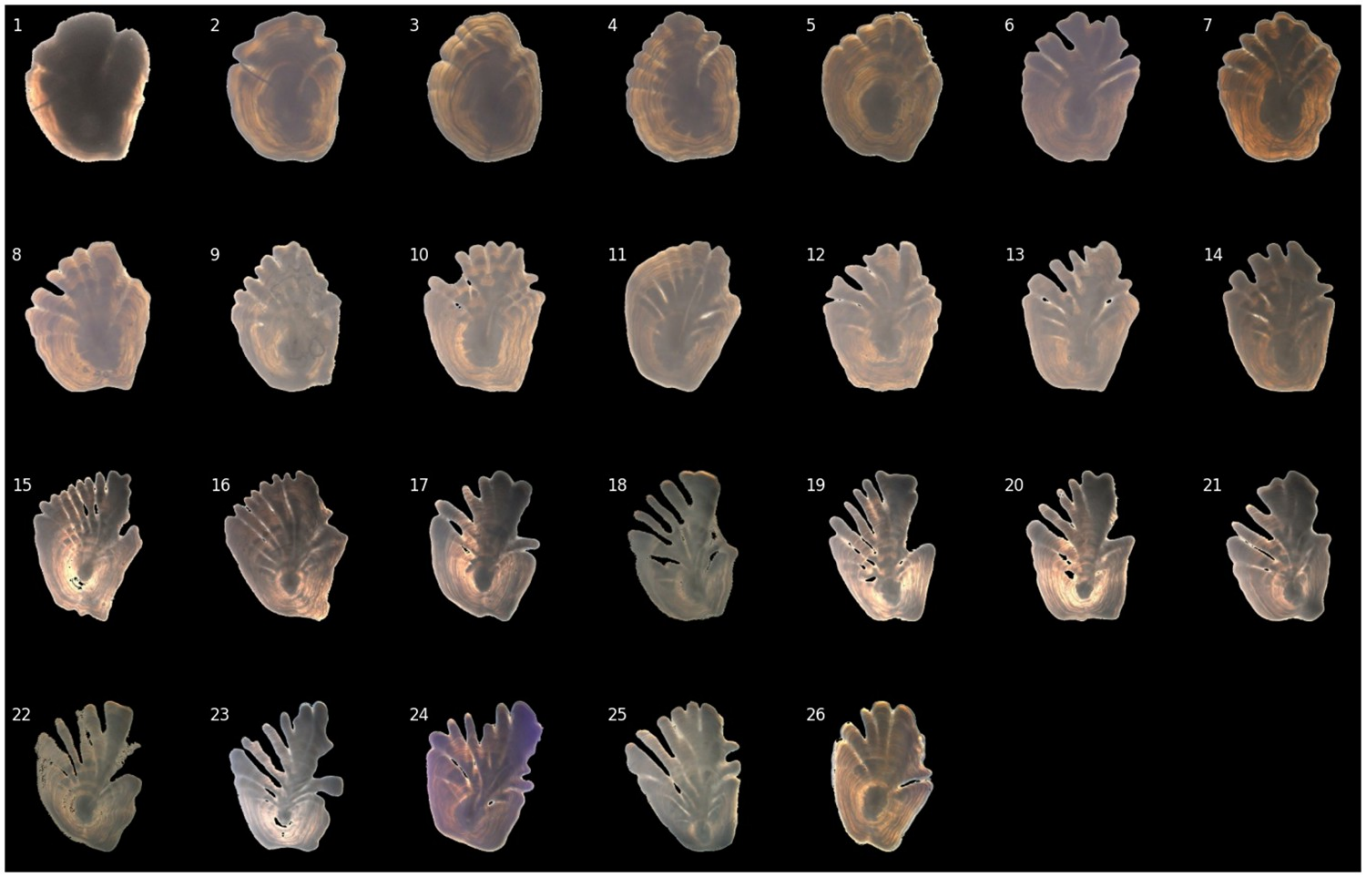
Selection of Greenland halibut otoliths. The figure shows a selection of Greenland halibut otoliths for each age in the range of 1 to 26 years. The images are standardized to have the same maximum vertical extension. The most evident growth pattern is the development of fingers and holes in the otolith as the fish grows older. The annual zones are not visible in all images. We see, however, that the dark initial core with a diameter of roughly 2 mm, has a relative location that gets lower with age, i.e., the upper part of the otolith grows faster with age than the lower part.

The age estimation in this paper is based on the analysis of images of translucent otoliths taken with backlight (Fig 1). The dataset is publicly available [11] and consists of 4,243 Greenland halibut otolith images in RGB format of size 600 × 600 pixels, all of which are associated with an age label in the range of 1 to 26 where the age is estimated (read) by marine biologists (readers). Two readers participated in the labeling of the data, and each image was read once by one of the readers. Both readers were experienced with a negligible between-reader bias. To avoid having a large model that could be overfit due to the limited set of data, images with a reduced size of 256 × 256 pixels were used to train the model. Fig 2 shows a selection of otoliths, where one image for each age group is selected. All images were standardized with respect to otolith height during preprocessing, i.e., all otoliths have the same maximum number of pixels across their vertical dimension. As discussed in Ref. [9], using standardized images encourages the network to focus on the visual pattern in the otoliths rather than on the otolith size. The otoliths change shape as the fish ages and the growth pattern, apparent when looking at the figure, suggests that both the length and the number of fingers are indicators of the age of the fish. From the figure, we see that the otoliths of juveniles (ages 1–4) have an approximate circular shape without fingers at all, while adolescents (ages 5–9) and young adults (ages 10–13) appear to have an early development of fingers in the upper half of the otolith. Fingers appear to be more prominent in adult fish (ages 14–26).

In addition to the age, sex and length measurements were also provided for 3,540 of the otoliths, which provides additional explanatory features that can be utilized in age prediction. In total, the data set consists of 1,465 images of male halibut (34.5%), 2,075 images of females (48.9%) and 703 images of halibut otoliths of unknown sex (16.6%). The dataset does not come with a predefined split in training, validation, and test sets.

**Fig 3:**
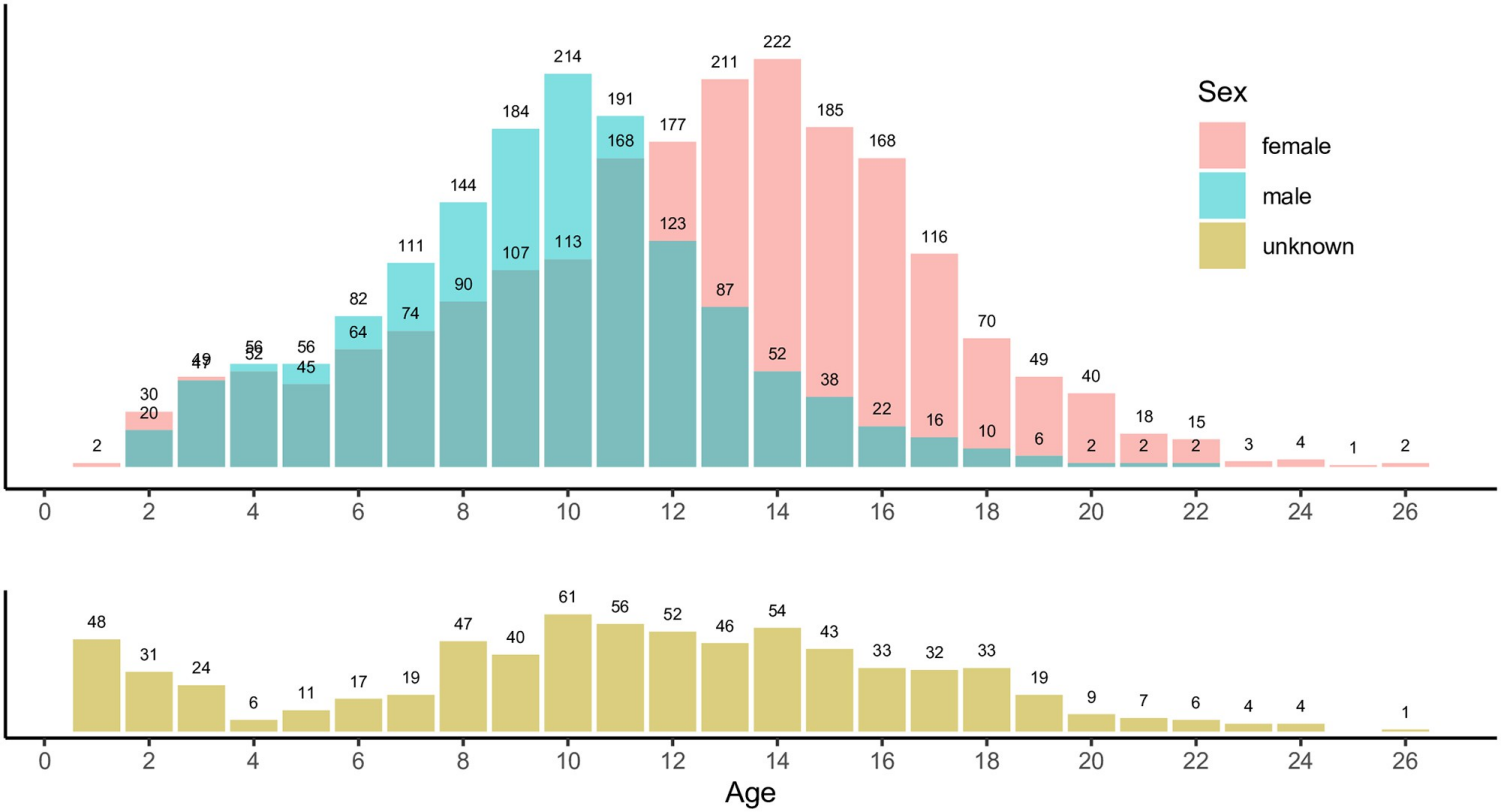
Age distributions for the data set. A bar chart showing the age distribution for the three groups (males, females, and unknowns). Female halibut have a longer lifespan than male halibut.

Fig 3 shows the age distribution for the three groups (males, females, and unknowns), where the distributions have rounded means of 10, 12 and 11 years, respectively, and medians of 10, 13, and 11 years. Standard deviations are 3.3, 4.4, and 5.4 years. The difference in the distributions of male and female ages is expected, as it is well known that female halibut live longer than males.

**Fig 4:**
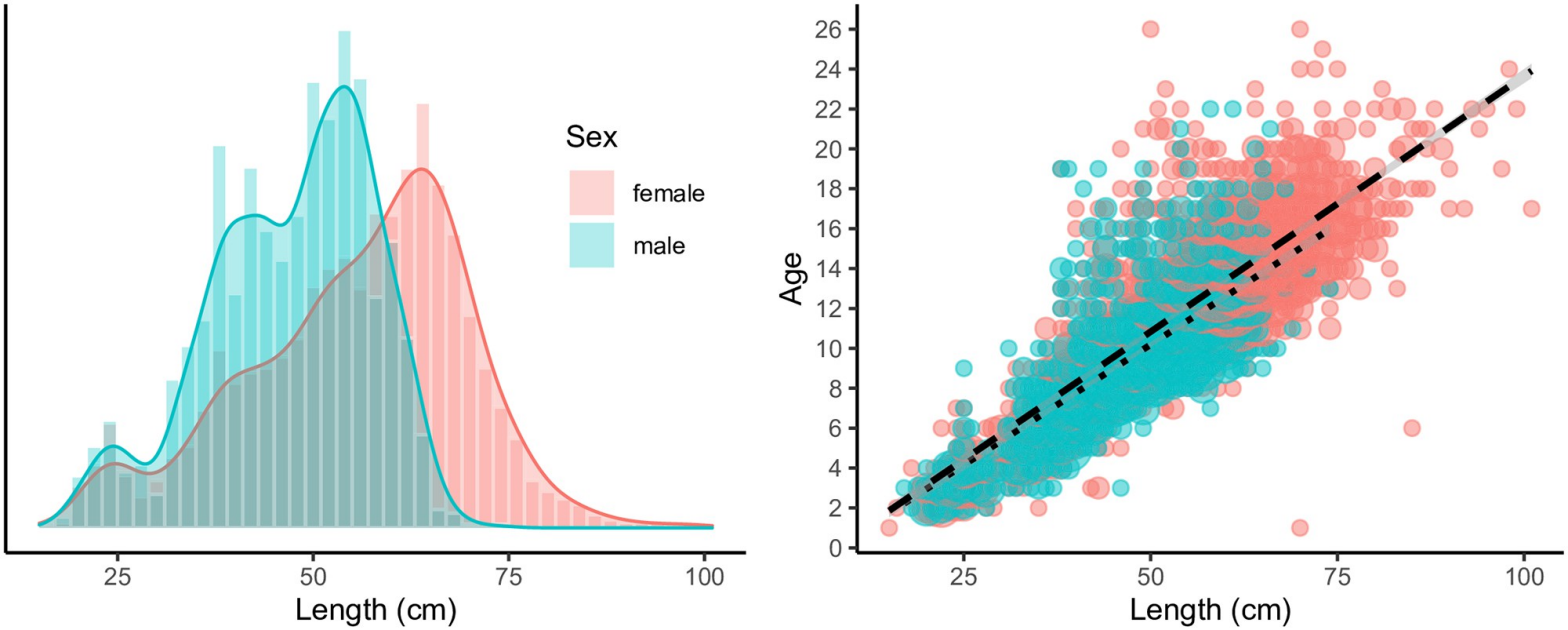
Age versus length. Left: Length distribution for the dataset. As for age, the female distribution has a larger mean as well as a larger variance than the male distribution. Right: A scatter plot of the labeled age against the measured length. The two dotted lines shows a fitted regression line for each sex.

The length distribution is shown in Fig 4A for all images in our dataset of known length. Since the length is a potentially relevant feature for the estimation of age, the relationship between age and length is often modeled by marine biologists by the well-known von Bertalanffy growth function [12]. For this data set, satisfactory results were achieved considering a linear relationship between the age and the fish length (Fig 4B, see S1 Appendix for details). In particular, a linear regression model that maps length to age represents a baseline that can be compared and combined with image-based deep learning estimates. The possible inclusion of length as an age prediction feature will be outlined in the Results section.

#### A note regarding read age uncertainty

The model in this paper is fitted with the objective of minimizing the discrepancy between the output of the model (predicted age) and the provided ground truth (read age). Even though we use the term “ground truth” when referring to the age labels, the true age remains unknown as the labels used in this paper are estimates from human experts. There is uncertainty regarding the accuracy and the precision of the human readers, which concerns the difference between the empirical labels and the actual age, and to what extent repeated readings by the same reader of the same otolith will give varying results. It is reasonable to assume that the precision in otolith age estimation will be worse with increasing age, but there is also a range of other possible sources of variations. Covariates that may influence readings include, e.g., image quality and seasonal differences.

There are ways to estimate the reader’s uncertainty:

- Accuracy can be assessed using a catch-recatch procedure in which an injected material is applied to the fish, placing detectable marks on the contour of the otolith (Fig 1A). When re-captured some years later, the true age difference between the mark on the otolith image and the outer contour is revealed.
- Precision can be assessed by comparing empirical labels given by several readers, as well as comparing repeated readings by the same reader. In cases where age estimation is performed by several readers, a typical coefficient of variation (CV) of about 12–14% is reported, but this measure is not directly comparable to the CV reported in this paper.

The estimation of reader accuracy is generally a fundamental challenge for many species.

### Proposed model for age prediction

When estimating the age of halibut using images, we assume that the age can be inferred by features appearing in the otolith images. In this case, the response variable is the age, *y*_*i*_, which is estimated using the corresponding otolith image, $X_{i}$. In addition, we include sex as a categorical variable, *z*_*i*_, which indicates if the image belongs to a male, female, or a fish whose sex is unknown. Fig 3 indicates that sex is an important factor in age distribution, though this has not been considered in previous Greenland halibut studies [8–10]. Sex information is readily available and routinely recorded when otoliths are extracted from the fishes. This leads to the regression equation in Eq (1), where ***θ*** denotes the unknown parameters of the regression function, and *ϵ*_*i*_ are random error terms. The function *f*(⋅) can be a deep learning model where ***θ*** are the model weights that are optimized during model training.

$$\begin{array}{c} y_{i}=f(X_{i},z_{i};\theta )+\epsilon_{i} \end{array}$$ (1)

As usual in regression, the mean squared error (MSE) in Eq (2) between the predicted age (${\hat{y}}_{i}$) and the read age (*y*_*i*_) is used as the loss function. The justification for using MSE instead of other regression metrics is provided in S1 Appendix.

$$\begin{array}{c} {\text{MSE}}_{\text{loss}}=\sum_{i=1}^{n}{\left(y_{i}-{\hat{y}}_{i}\right)}^{2} \end{array}$$ (2)

As a remark, we notice that it would be possible to classify the otoliths, for instance, into classes of juveniles (age 1–4), adolescents (6–9), young adults(10–13) and adults(14+) [7, 9, 10]. However, we frame our problem as a regression task since we assume that there exists an inherent relationship between images that are close in age. In this way, we can take advantage of the fact that similar otolith images should be associated with similar fish ages.

### Neural network architecture and transfer learning

To regress images into ages, we need a function *f*(⋅) (Eq (1)) that is able to handle complex inputs. This can be achieved by CNNs, which are particularly suitable for processing image data. CNNs have a complex architecture, which can be designed in different ways. Examples of popular architectures are AlexNet [13], VGG-16 [14], Inception [15, 16], and Xception [17], which are optimal for different types of task and datasets.

For the estimation of the halibut age, we selected the Xception architecture as our model. The Xception architecture is an evolution of the Inception models and has been shown to slightly outperform models such as Inception v3—used in previous works focused on fish age prediction [6–8]—on datasets such as ImageNet [18]. Both Inception and Xception have a relatively small number of parameters compared to other CNN architectures, which make the models suitable for our task since the otolith data set is relatively small. The main characteristic of the Xception architecture is the utilization of depthwise separable convolutions that, compared with the standard convolutions (S1 Appendix) used by other networks, reduces the number of parameters in the model by decoupling the convolutions into a depthwise and a pointwise step. The network design also includes residual skip connections [19] between blocks, which are known to facilitate training in very deep architectures (S1 Appendix).

We used pre-training, which means that the model weights (***θ***) fitted to the ImageNet database [18] were used as initial parameters. Using a pretrained model (a technique known as transfer learning) often provides a good starting point for fine-tuning the model since low-level feature detectors (e.g., edge detectors) can be inherited from one task to the other. In addition, pre-training can improve the generalization capabilities, especially when dealing with datasets of small/medium size [20]. For this particular task, we observed that fine-tuning a model pre-trained on ImageNet with more than 20,000 classes was more efficient than training a model from scratch on the otolith data.

#### Model schematics

**Fig 5:**
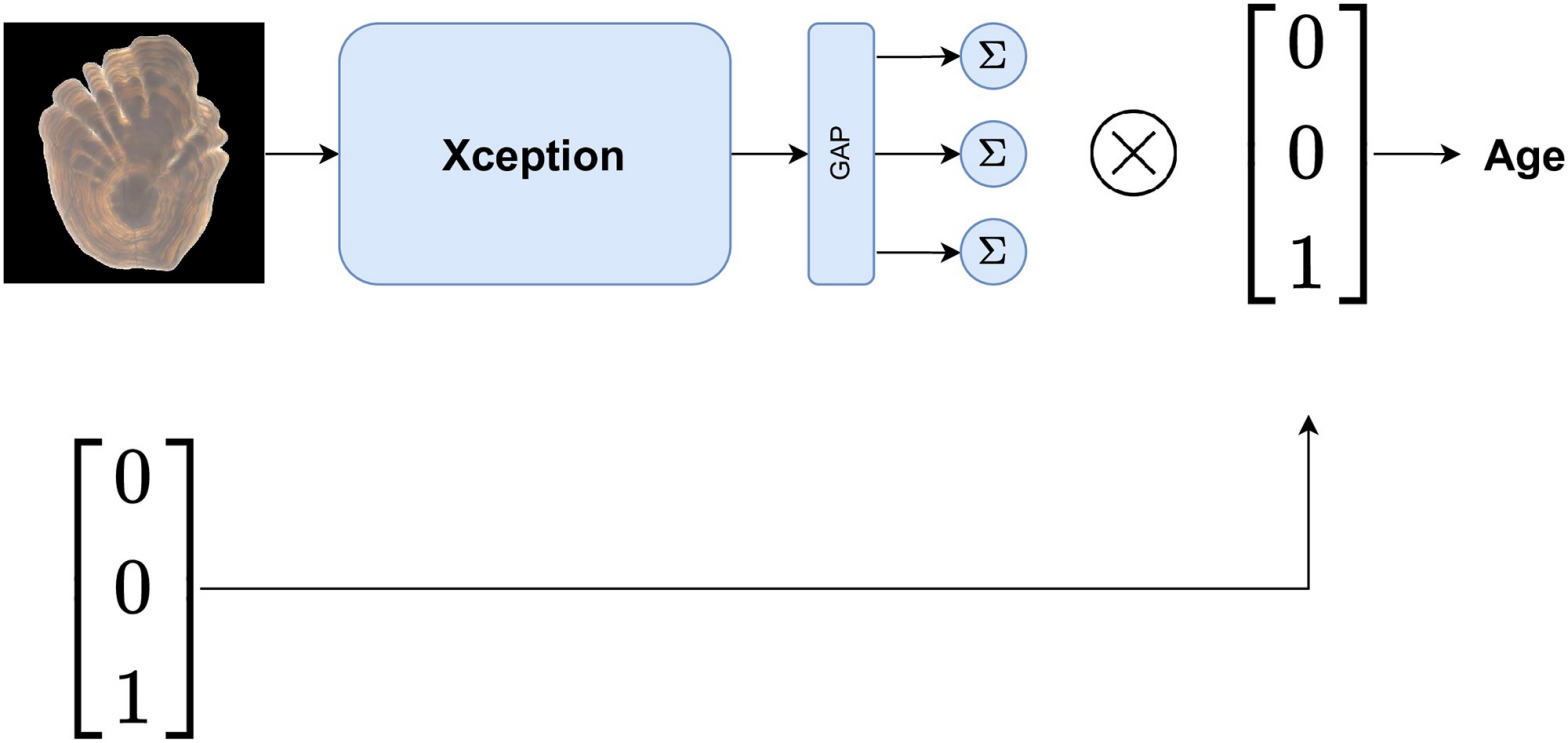
Model outline. Schematic look of the age estimation model. The *Σ*-nodes gives different age estimates conditioned on the sex of the input.

Fig 5 shows the outline of the image prediction pipeline where the image is processed by the Xception CNN architecture followed by a global average pooling layer (GAP) [21] and a dense layer with three output nodes (*Σ*). The three output nodes are equipped with a ReLU activation function (Eq (3)), which ensures that the predicted age is a positive real number.

$$\begin{array}{c} f\left(x\right)=\{\begin{array}{cc} x & ,x>0\\ 0 & ,x<0 \end{array} \end{array}$$ (3)

GAP converts the Xception output into a vector, which is then passed to each of the output nodes, which in turn takes a weighted sum of the GAP vector output, passes it through a non-linear activation function and returns a scalar. The three output nodes, each mapping the input into a positive real number, represent the conditional age estimation given that the sex corresponding to the otolith image is male, female, and unknown, respectively. Thus, by taking the dot product of the output with a one-hot encoded vector representing the sex, we can obtain the final age estimate. A one-hot encoded vector is a categorical vector with exactly one element equal to one, and the rest equal to zero. A male halibut is encoded as [100]^*T*^, a female as [010]^*T*^ and an unknown sex as [001]^*T*^.

The proposed layout of the neural network architecture consists in a common feature extractor for the images of all sexes and an output layer specialized in predicting the age for each sex. Having a separate output for each sex allows us to directly account for the sex, which is given as prior information. This would not be possible in a model with a single output for all otoliths. The proposed architecture is also preferable to fitting one model for each sex, as such models can be trained only on a fraction of the whole dataset (i.e., only images of male or females). Finally, since we assume that there are common age-indicative features between the three groups, it is reasonable to have a shared feature extractor for all sexes. For instance, male predictions can benefit from features learned from female data.

### Strategies for model training

The deep learning model was trained using numerical optimization based on minimization of the loss function in Eq (2). Since deep learning models are prone to overfitting, several regularization methods were used to improve the generalization capability of the model. The depthwise separable convolutions in the Xception architecture and the downsampled input images contribute to reducing the number of parameters in the model. We also applied an L2-regularization on the model weights, which penalizes the model complexity by increasing the loss if the parameters get too large in magnitude. We also used dropout [22] between the GAP layer and the output nodes (Fig 5). Dropout is a popular regularization technique that, by randomly dropping nodes during training, simulates an ensemble of different network architectures, which is known to reduce overfitting.

**Fig 6:**
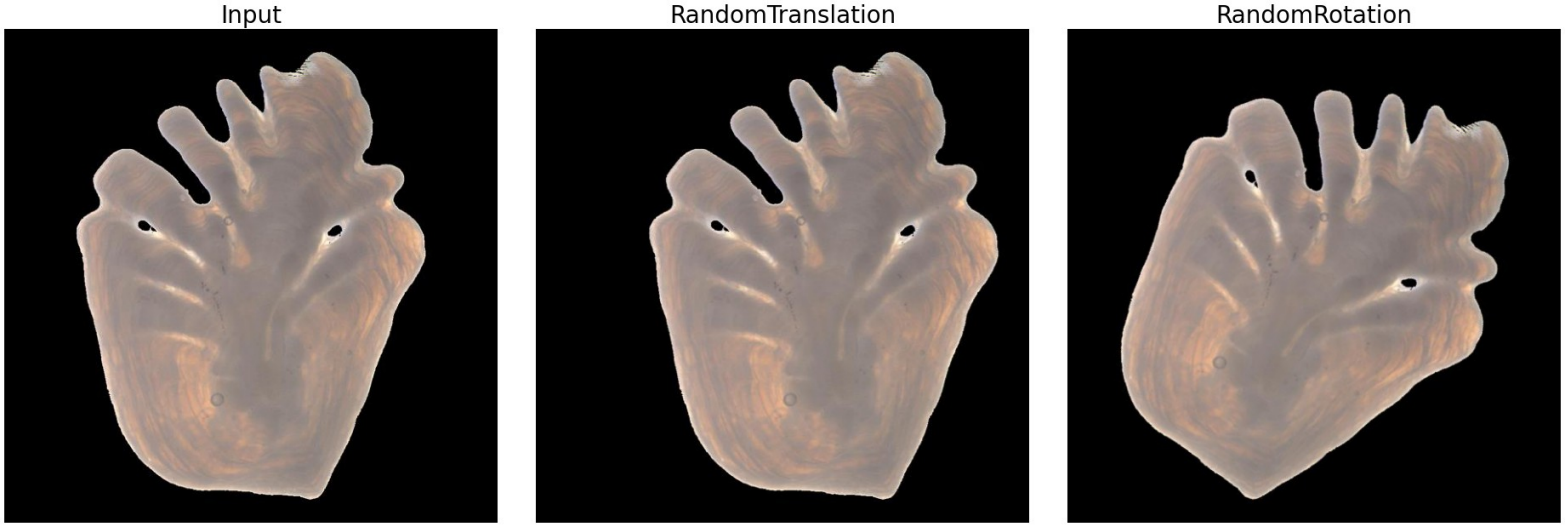
Data augmentation. Random translation and rotation applied on an otolith image.

To further increase the generalization capabilities of the model, data augmentation was used by randomly translating and rotating the input images. Only horizontal translation with a 10% range was used, since the input images were standardized with regard to height. The rotation was applied using a factor of 0.1 (maximum rotation of 36°/0.2*π* in both directions) (Fig 6).

The data set was divided into training, validation and test data, where the loss was monitored on the validation set, and where the weights that achieved the best performance on the validation set were chosen as model parameters. This was achieved by early stopping with a patience parameter of 20 and a maximum number of epochs of 100, which means that we stopped training if 20 epochs passed without improvement in the validation loss. Images with unknown sex (703 in total) were only used for training, while 10% of the remaining images were chosen for both validation and testing (354 images for both sets). The images were randomly sampled using proportionate stratification based on read age, so that the age distribution was approximately equal for all three splits (see S1 File for the Python code).

The parameters in the batch normalization layers were frozen during training to avoid ruining the patterns learned during pre-training, while all other parameters were trainable. The initial bias for the three output nodes was set equal to the mean ages of the three groups (males, females, and unknowns) to accelerate convergence.

The model parameters are optimized using gradient descent, where several variations of the algorithm (optimizers) exist. Examples of optimizers are Adam [23], Adagrad [24], AdaDelta [25] and RMSprop. In this work we adopt Adam, which is often considered to be the best default choice in the recent deep learning literature [26]. The Adam optimizer was also used in previous related work focused on the age estimation of Greenland Halibut [8, 9], and the analysis of fish otoliths belonging to other species [7, 10].

#### Hyperparameter tuning

We searched the optimal dropout and learning rates through a random search with KerasTuner [27]. For training, we used images with unknown sex (703) in addition to 80% of the remaining 3540 images with known sex, while 20% of the images with known sex were used for validation. The dropout rate was sampled linearly from 0 to 0.5 with steps of 0.1, while the learning rate was sampled from the range 1e-1 to 1e-6 using logarithmic sampling. The lowest MSE loss out of 20 attempts was 2.97, achieved with a dropout rate of 0.4 and a learning rate of 10^−4^. The other hyperparameters were selected by hand. All hyperparameters are summarized in Table 1.

**Table 1:** Hyperparameters.

Model details and hyperparameters	
Input shape	(256, 256, 3)
Data augmentation	Horizontal translation and rotation with a limit of 10%
Batch size	32
Output initial bias	9.52, 12.32, 11.07
Output activations	ReLU
Loss function	mean squared error
Optimizer	Adam optimizer
Learning rate*	1e-4
Weight of the L2 norm of the parameters	1e-5
Dropout rate*	0.4
Patience	20
Max epochs	100

A summary of model details and hyperparameters. Hyperparameters with * are optimized with random search.

### Cross-validation

It is necessary to perform a cross-validation procedure to be sure that the model performance truly generalizes to independent data. Leave-one-out cross-validation is a well-known method that is very unpractical for neural networks, as the model fitting procedure is time-consuming and computationally expensive. Options more frequently adopted when working with neural networks are *k*-fold and k*l-fold cross-validation, which are exhaustive in the sense that every image is at least once part of the test set.

In k*l-fold cross validation, the dataset is divided into *k* subsets, where each subset is used as a test set once. The data included in the *k—1* remaining subsets are further divided into *l* subsets, each of which is used as a validation set once. Finally, the model is fitted on the training set consisting of the remaining *l—1* subsets. This method results in k*l different data splits, each example being tested *l* times.

In our training procedure, a variation of k*l-fold cross-validation using early stopping was used, where the set of data with known sex (3540 images in total) was split into 10 subsets of size 354 using proportional stratified allocation based on read age. Each subset was chosen once for testing, while one of the remaining nine subsets was used for validation. The training set consisted of the remaining 8 subsets, in addition to the images where the sex was unknown. Alg 1 shows the details of the cross-validation procedure.

**Algorithm 1** A variant of k*l-fold cross-validation algorithm with Early Stopping

$D=\{D_{1},\ldots ,D_{10}\}$

**for** *i* in 1 to 10

 $D^{*}=D\backslash \left\{D_{i},D_{i+1}\right\}$

 Obtain *f*_*j*_ (trained network): function fitted on $D^{*}$ using early stopping to $D_{i+1}$

 Obtain *e*_*j*_ (loss): *f*_*j*_ performance evaluated on $D_{i}$

**end for**

### Evaluating test results

A useful metric to evaluate the performance in the estimation of fish ages is to measure whether the distribution of the predicted age closely resembles the actual age distribution. Despite its importance, we notice that such a measure was not reported in previous related studies [6–10]. The closeness between two distributions can be measured by computing the Kullback-Leibler (KL) divergence between the predicted density (*Q*(*x*)) and the density function of the ground truth (*P*(*x*)) following the formula in Eq (4). In our case, since the parametric age distributions are unknown, they are approximated using kernel density estimation and the KL-divergence is computed using numerical integration (see S1 Appendix and S1 File for details).

$$\begin{array}{c} D_{KL}\left(P||Q\right)=E_{X∼P}[\text{log}\frac{P(x)}{Q(x)}]=E_{X∼P}[\text{log}P(x)-\text{log}Q(x)] \end{array}$$ (4)

Other common metrics evaluate performance relative to individual age estimates. The root mean squared error (RMSE) between the ground truth (read age) and the predicted age is the most natural to use, as the unit is in years and the model is optimized with respect to the MSE. Another metric that has been adopted in related works [8–10] is the mean coefficient of variation (CV). The CV of an example image *i* is calculated using the ground truth ${\hat{y}}_{i}$ and the predicted age *y*_*i*_ using the formula in Eq (5) where ${\bar{y}}_{i}=\frac{{\hat{y}}_{i}+y_{i}}{2}$. The mean CV is then computed across all test examples (Eq (6)).

$$\begin{array}{c} {\text{CV}}_{i}=\frac{\sqrt{{\left({\hat{y}}_{i}-{\bar{y}}_{i}\right)}^{2}+{\left(y_{i}-{\bar{y}}_{i}\right)}^{2}}}{{\bar{y}}_{i}} \end{array}$$ (5)

$$\begin{array}{c} \bar{\text{CV}}=\sum_{i=1}^{n}{\text{CV}}_{i} \end{array}$$ (6)

We also consider two additional metrics: the percentage of test images where the rounded age estimates are equal to the ground truth (0-off percentage), and the percentage of test images where the predicted age is at most 1 year off the ground truth (1-off percentage).

### Explanation techniques

Deep Learning models are often considered black boxes, and there exists examples where the decision rules of a model are based on image artefacts or biases that appear in the training set [28]. To trust the model’s generalization capabilities it is, therefore, useful to analyze the relationship between the input features and the model output. A model is inherently interpretable if the relationship between input and output can be easily understood and explained by human reasoning [29]. However, this is not the case for neural networks, which are black boxes, and we must resort to heuristic techniques to explain and interpret the decision of a trained model. For example, a way of gaining insights into the model’s decision process is by analyzing the relevance of the input features to the model output. There exist several methods for quantifying the relevance of input features, all of which have in common that they attribute scores to the features based on how much they contribute to the model decision.

There exist several methods for attribution of relevance scores to input features, and these methods differ in their approach and in their final result. The attribution methods that we chose to apply for the Greenland halibut otolith images are gradient saliency maps [30], baseline gradients, integrated gradients [31], guided backpropagation [32] and integrated guided gradients. These methods are described in S1 Appendix. Gradient saliency maps and guided backpropagation use relevance scores that are equal to the gradient of the output with respect to each input pixel. Pixels corresponding to high gradients are considered relevant as a small change in these pixels would result in a big change in the output. Baseline gradients, integrated gradients and integrated guided gradient are gradient based methods that compare the output obtained by a *non-informative baseline* and the actual image, to establish which are the most important input pixels.

#### Choosing the right non-informative baseline

Choosing the right non-informative baseline image is important to obtain informative results. The baseline image should be non-informative in the sense that the classification of a baseline image should not be biased towards a particular class. Natural baselines include all-black images, all-white images, or images with randomly sampled pixel values. A common problem is that predicting baseline images often results in biased predictions, i.e. that the baseline image automatically gets assigned to the majority class, or as in our case, assigned with a predicted label equal to the mean response of the data. A proposed suggestion to counteract this effect [33] is to add another class to the data set consisting only of baseline images to prevent biased baseline predictions. Using such an additional fake class, ensures that the score for the baseline is close to zero for all other classes. The approach taken in this paper is to add 50 black baseline images and label them with age equal to zero.

### Second age estimates using length

In addition to predicting age using otolith images, we also obtained a second age estimate by performing linear regression on length and sex. This was done both to compare deep learning predictions with a simpler method, and to understand if adding length as an additional feature would increase the predictive power of the model. The length-based age estimates were obtained by fitting the linear regression model in Eq (7), where *Y*_*i*_ and *x*_*i*_ are the age and length of the observation *i*, and *z*_*i*_ is an indicator variable equal to 1 if the observation *i* is male. The intercept parameter *β*_0_ is assumed to be equal for the two sexes, since previous studies showed that male and female fish have the same expected length at birth [34]. The results were calculated on the exact same test sets described in the Cross-validation section, but the regression model was fitted to both the training and validation data.

$$\begin{array}{c} E\left(Y_{i}\right)=\beta_{0}+\beta_{1}x_{i}+\beta_{2}x_{i}z_{i} \end{array}$$ (7)

## Results

All deep learning code is written in Python [35] using the Keras API [36]. The TensorFlow framework [37] is used for model fitting, while R [38] is used for analysis and figures. The source code and notebooks used in this work can be found at https://github.com/IverMartinsen/MastersThesis.git.

### Model performance and fit

**Fig 7:**
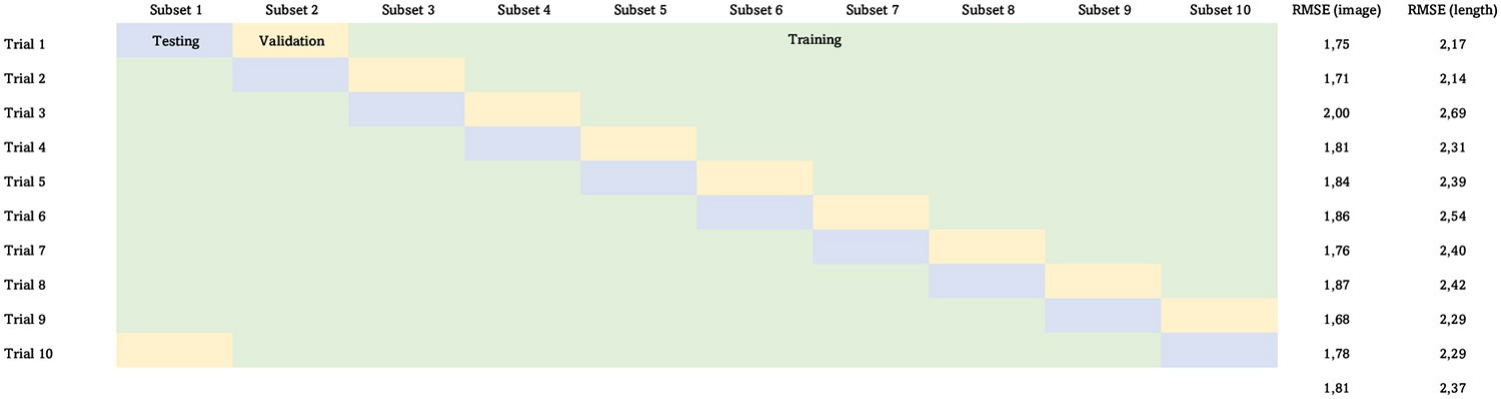
Cross-validation test results. Individual test results for all cross-validation trials for both the image based deep learning age estimates, and the linear length based age estimates.

Fig 7 shows the test results for both the image based deep learning predictions, and the linear length-based predictions, where the cross-validation procedure was performed with 10 disjoint partitions of the data set. We clearly see that test results vary to a large extent based on the split, where the RMSE loss varies from 1.68 to 2.00 for the image based age estimates, and from 2.14 to 2.69 for length based estimates. In Table 2, the summary results are reported for each sex in terms of RMSE, mean CV, 0-off percentage and 1-off percentage, where we see that deep learning predictions are clearly better than predictions based on length regression. Overall, the results are better for male halibut compared to female halibut, which is expected since the female age distribution has a larger spread than the male distribution (Materials and methods—Greenland Halibut otolith data).

**Table 2:** Summary of test results. Summary of overall test results for age estimation using deep learning and linear length-based regression.

	RMSE			mean CV			0-off percentage			1-off percentage		
	Males	Females	Total	Males	Females	Total	Males	Females	Total	Males	Females	Total
Deep Learning	1.58	1.95	1.81	9.55%	9.44%	9.48%	27.6%	21.8%	24.2%	70.0%	60.4%	64.4%
Length regression	2.21	2.48	2.37	12.37%	11.58%	11.90%	21.6%	18.5%	19.8%	59.2%	53.2%	55.7%

**Fig 8:**
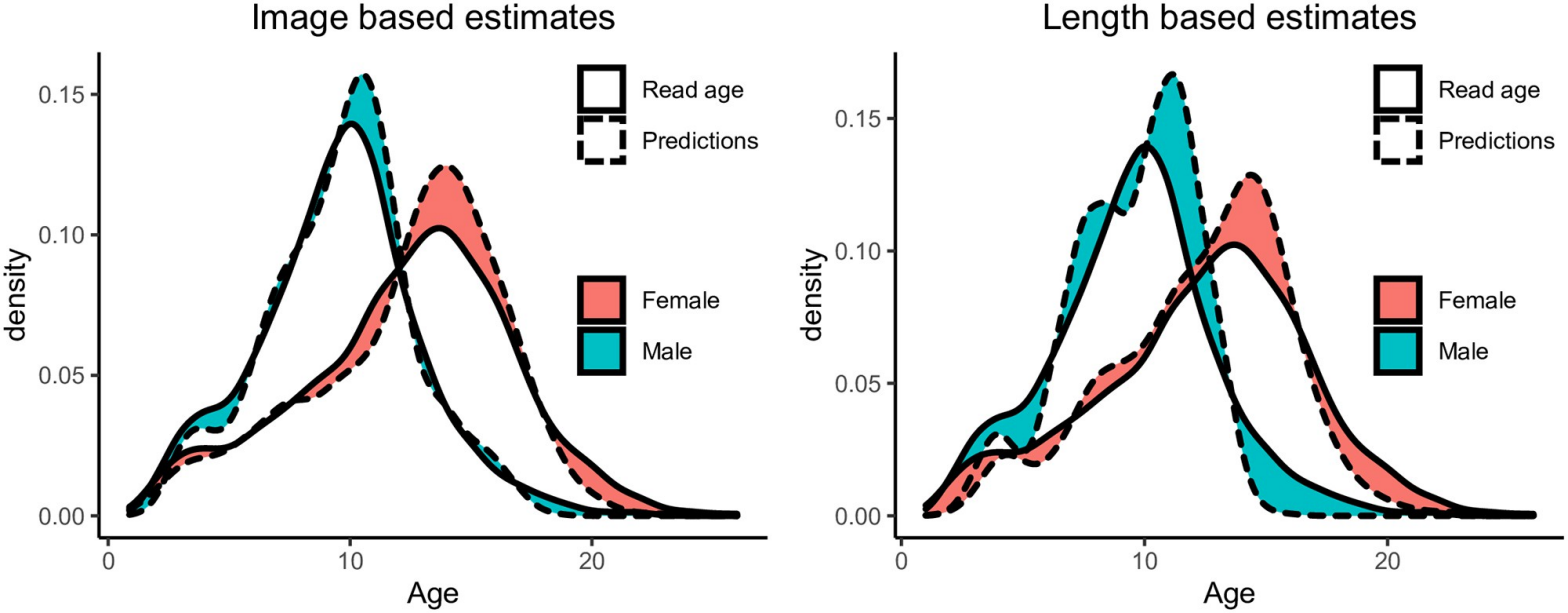
Comparison of age distributions. The figure highlights the difference between the distribution of the read age and the predictions. A: Density estimates for image based predictions. B: Density estimates for length based predictions.

As previously discussed, it is important that the distribution of the predictions is close to the true age distribution. Fig 8 shows a comparison of the estimated density for the ground truth (read age) and the age predicted by deep learning (Fig 8A) and linear regression (Fig 8B), where the shaded region highlights the difference between the estimated density curve for the ground truth (solid line) and the predictions (dotted line). The deep learning age predictions are distributed similarly to the ground truth with a KL-divergence of 0.02 for the male age distribution (Table 3), and 0.03 for the female distribution. The length-based estimates have a particularly poor fit for male halibut (0.17).

**Table 3:** KL-divergence. KL-divergence of the predicted age distributions with regard to the distribution of the ground truth.

	Males	females
**Deep learning predictions**	0.02	0.03
**Length regression**	0.17	0.07

### Bias and standard deviations

**Fig 9:**
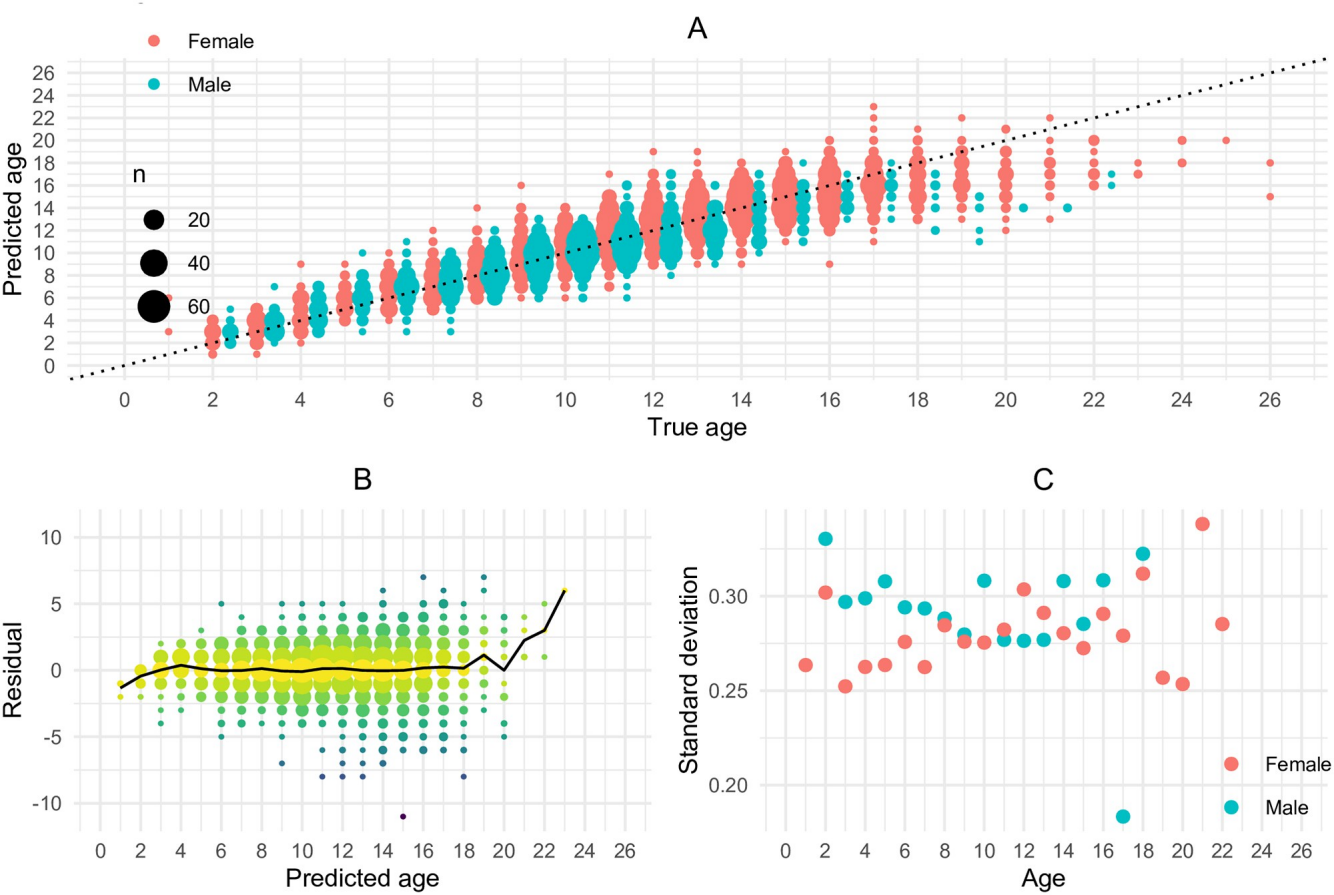
Scatter plot of predictions and residuals. A: Scatter plot of the predicted age against the ground truth (read age). B: Residuals plotted against predicted age (solid line shows average residuals). C: Empirical standard deviation for the residuals plotted against read age. The standard deviations are computed for each age, using the residuals corresponding to that age as the sample.

Fig 9A shows the predicted age vs the ground truth (read age) and indicates that the deep learning age estimates seems to be unbiased from the age of 3 to 12 for males and from the age of 3 to 15 for females. The model underestimates the age for older fish. This is expected because the otoliths of older fishes exhibit more complex features and because there are fewer examples of old fishes in the data set. The results do not display any sign of significant heteroscedasticity (Fig 9B and 9C). The spread of the residuals in Fig 9A seems to be largest around the mean age. However, since the sample is also largest for these ages, there is a larger probability to observe extreme values that gives a false impression of larger variance. In fact, when we compute the empirical standard deviation of the residuals, they seem to be approximately constant (Fig 9C). The standard deviations in Fig 9C are computed for each age, using the residuals corresponding to that age as the sample.

### Including length as an explanatory feature

Although image-based estimates and length-based estimates have a high correlation (0.86), it is possible to combine them with the aim of improving predictions. To examine whether adding length as a feature brings improvements, we define a new age estimate computed as follows:

$$\begin{array}{c} {\hat{y}}_{i}=\alpha {\hat{y}}_{1i}+\left(1-\alpha \right){\hat{y}}_{2i}, \end{array}$$ (8)

where the combined estimate ($\hat{y}$) is a weighted sum of estimates based on images (${\hat{y}}_{1}$) and estimates based on length (${\hat{y}}_{2}$). The parameter *α* is obtained by fitting Eq (8) using linear regression, resulting in a *α* = 0.80, which we use to obtain the fitted values for Eq (8). The fitted values resulted in an RMSE of 1.76, which is slightly lower than the RMSE of 1.81 obtained by deep learning model only trained on the images (Table 4). However, the KL-divergence, was higher for the combined predictions, with a value of 0.04 for males and 0.06 for females. The results are summarized in Table 4.

**Table 4:** Results after including length-based predictions as a second age estimate. Summary results for image-based estimates (deep learning predictions), and a combination of image-based and length-based estimates (combined predictions).

	RMSE	KL-divergence, males	KL-divergence, females
**Deep learning predictions**	1.81	0.02	0.03
**Combined predictions**	1.76	0.04	0.06

### Explaining decisions by heatmaps of pixel relevance

**Fig 10:**
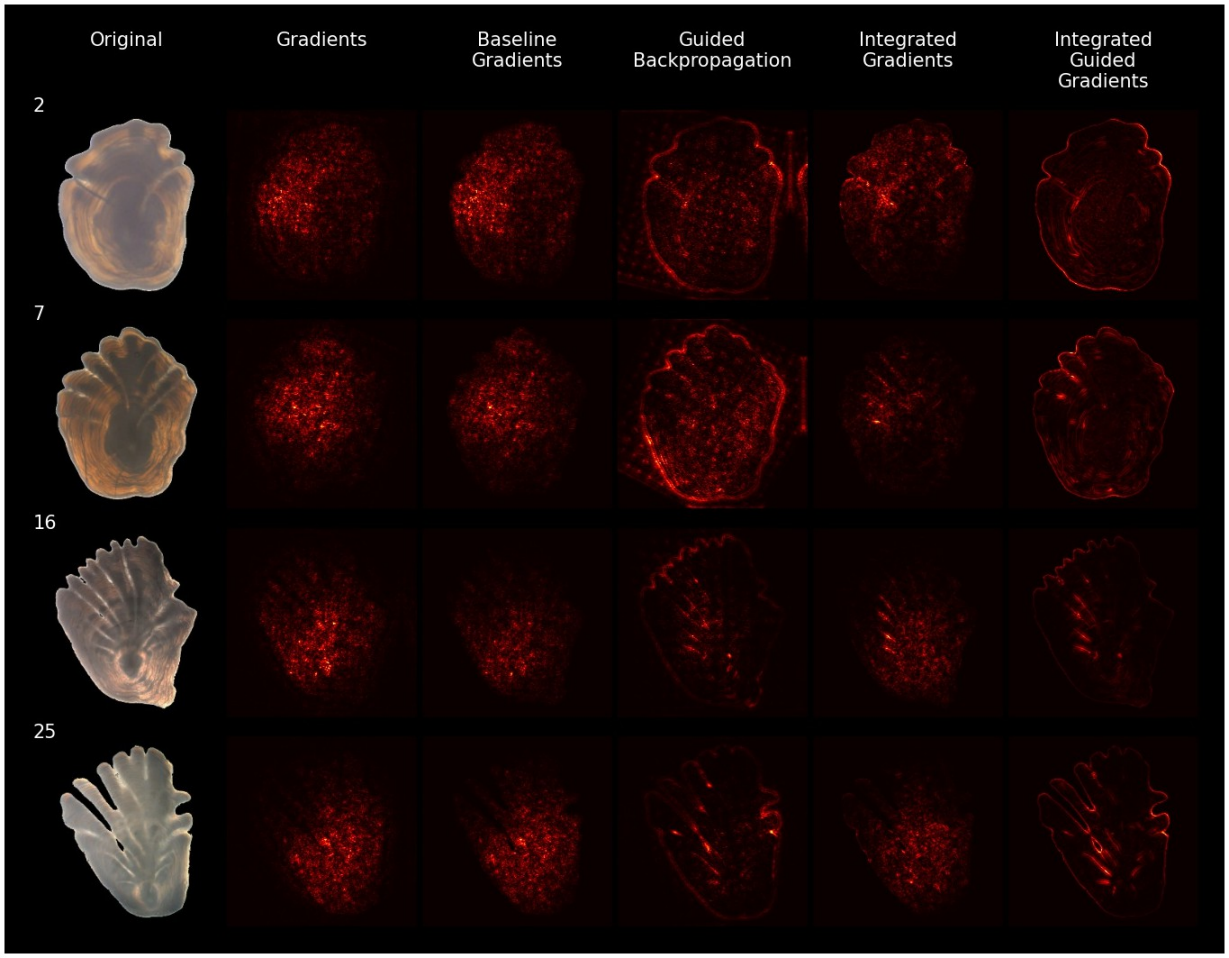
Comparison of explainability methods. Heatmaps produced by five different techniques, for a selection of otolith images from different age groups.

We applied different methods to quantify the relevance of the input features. Relevance is displayed using a heatmap that highlights how much each input pixel contributes to determine the output. Fig 10 shows a comparison of five different methods used to estimate the relevance of pixels across a selection of four different input images. The heatmaps produced by the different methods are not identical since they are heavily influenced by the specific heuristic followed by each explainability method.

From Fig 10, we see that the baseline gradient heatmaps and the gradient saliency maps are noisy and difficult to interpret, while the guided backpropagation heatmaps differ from the others in that the contour of the otolith is emphasized, an effect that is especially noticeable in the first two images (2 and 7). The integrated gradient heatmaps contain fewer bright spots than the heatmaps produced by gradients, whereas the integrated guided gradient heatmaps appear very similar to the heatmaps produced by guided backpropagation. The difference between the two methods is noticeable in the first two images, where the integrated guided gradients produced smoother heatmaps with less noise.

**Fig 11:**
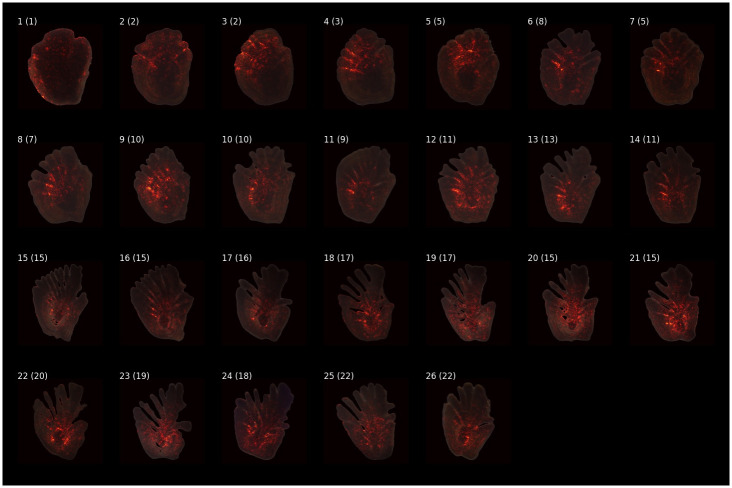
Feature relevance heatmaps produced by integrated gradients. The integrated gradients technique is applied to a selection of otolith images. The number in the parenthesis displays the age predicted by the deep learning model.

Fig 11 displays heatmaps produced by integrated gradients applied to the selection of otoliths shown in Fig 2. Even though the annual zones are considered the most important part of the otolith as they are counted by the readers to determine the age, those are not particularly emphasized in the resulting heatmaps. This is expected, as annual zones are extremely difficult to count on low-resolution images, especially as the age of the fish increases. Image features that are emphasised as important by the heatmaps are the fingers (stripe-like pattern apparent from the age of 3 to 16) and the core of the otolith, which is highlighted in otolith images of older fish (age of 9 to 26). As previously mentioned (Materials and methods—Greenland Halibut otolith data), the relative size of the core decreases with age and it is reasonable to assume that both the length and the prominence of the fingers in addition to the relative size of the core, are important in determining age from otolith images.

## Conclusions and discussion

Otoliths of Greenland halibut are visually analyzed by human experts and play an important role in determining the fish age. Otolith analysis is conducted extensively worldwide and the process is time-consuming both in terms of the analysis itself and in terms of the time used to educate the readers. In this paper we proposed an automatic procedure based on CNNs—a class of deep learning models used in computer vision—for predicting the fish age from an image of the otolith. The proposed procedure estimates the fish age consistently, in the sense that a trained CNN would produce the same output for the same input, which is a major advantage in comparison to estimates provided by human readers, which are often inconsistent. The results we obtained show that the deep learning approach has great potential. The most important observation is that the age distribution of the Greenland halibut predictions matches the distribution of the ground truth labels very well.

Previous related studies have achieved good results on both Greenland halibut [8–10] and other species [6, 7]. Our results are very similar to the results obtained in the previous studies focused on Greenland halibut [8–10], although two of these studies relied on a single test set [8, 9]. As we observed significant variations between the results obtained on different test set splits, the cross-validation procedures conducted in this paper demonstrated the importance of using robust techniques to validate model performances. For instance, the result obtained in [9] are slightly better than our average RMSE and CV, but worse than the RMSE and CV obtained on some of our splits. We also note that the study in [9] indicated that using standardized images slightly worsened the predictions for some age groups and that improved performance could be achieved using unprocessed images.

Length measurements are reliable and easily obtained and could be included as an additional feature to improve age predictions. Since it is known that the otolith continues to grow for the entire lifespan of the fish, one could argue that all information provided by length measurements should be contained in the otolith images and that length is a redundant feature. Combining deep learning estimates with age estimates did improve the performance in terms of RMSE. However, including the length also led to a slightly larger discrepancy between the distributions of the predicted ages and the ground labels, in terms of KL divergence. This is clearly undesired, as the overall age distribution is very important in stock assessment. Another well-known technique to use length measurements in age estimation is to establish so-called age-length keys (ALKs) [39]. By stratified sampling of the fish to provide otoliths by, e.g. measuring a given number of individuals per length group, such an ALK can be provided. Then one can combine ALK with a much larger (and cheaper) length-sample to obtain a more precise age-distribution. It should be straightforward to combine ALKs with deep learning as a possible direction for future work.

In many practical applications, it is important to understand the CNN decision process both to gain additional insights about the data and to check the validity of the model. For this purpose, we considered several explainability techniques for deep learning models that quantify the pixels’ relevance on the output in the form of heatmaps. While these tools can be useful in understanding the decision process of a deep learning model, they are mostly based on heuristics and the interpretation of the heatmaps is subjective. In our case, the heatmaps were difficult to interpret, as there were few patterns that provided clear and obvious explanations behind the model output, which may be due to both the complex decision rules derived by the deep learning model and the lack of strong characterizing features in the images. A possible reason is that since different images exhibit different characteristics, there are few strong patterns common to all images of the same age group. An image with a few distinct characteristics might result in a heatmap with sparse bright spots that is easy to interpret. On the other hand, an image with several weak characteristics might result in a noisy heatmap, since the model makes its decision by combining many features together. Greenland halibut otolith images belong to the latter category, as the features present in the images were only weak indications of the otolith age. Nevertheless, we identified a few interesting patterns in the resulting heatmaps. The development of fingers as the otolith grows and the relative size of the otolith core are clear indicators for fish age, both of which were emphasized by the heatmaps as stripe-like patterns around the core. This detail was not visible in [9], where the heatmaps was produced by averaging activations across age groups, resulting in smoother, but less detailed heatmaps.

The success of deep learning methodology to predict the age of fish from otolith images should also motivate the implementation of more appropriate imaging technology on board where the otoliths are sampled. One could think of a simple system with a robust camera mounted where the fish samples are taken such that the otolith could be photographed and labeled immediately. As good results are obtained with low-resolution images, the cost of such equipment would probably be acceptable and, potentially, could also be used to discriminate between different species as well as stocks.

## Supporting information

S1 Appendix Appendix. Additional information and analysis.
(PDF)

S1 File Python code. Function to produce indices for data splitting using proportionate allocation stratification.
(PY) Click here for additional data file.

S2 File R code. Functions for computing the KL-divergence using numerical integration and kernel density estimation.
(R) Click here for additional data file.
