# Supplementary material for: Age prediction by deep learning applied to Greenland halibut (Reinhardtius hippoglossoides) otolith images
Source: PLoS One. 2022 Nov 4;17(11):e0277244. doi: 10.1371/journal.pone.0277244 (PMC9635702; doi:10.1371/journal.pone.0277244)
Supplement: S1 Appendix — Additional information and analysis. (PDF) [file pone.0277244.s001.pdf]

Appendix

A Definitions

A.1 Mean squared error (MSE)

The MSE is defined in Eq (1) as the expected square difference between an estimator $\hat{\theta}$ and a parameter θ.

$$\mathrm{MSE}\left(\hat{\theta}\right) = E\left[\left(\hat{\theta} - \theta\right)^2\right] \tag{1}$$

While computing the MSE requires the knowledge of the distribution of the estimator $\hat{\theta}$, the MSE loss function defined in Eq (2) is concerned with computing the observed loss between a sample and a set of corresponding estimates. The MSE loss is calculated for a set of predictions $\{\hat{y}_i, i = 1, \ldots, n\}$ with respect to a set of target values $\{y_i, i = 1, \ldots, n\}$. It can be shown that under mild conditions [14], the minimization of the MSE loss function by gradient descent with decreasing learning rate converges to the minimum of the expectation.

$$\mathrm{MSE}_{\mathrm{loss}} = \frac{1}{n}\sum_{i=1}^{n}\left(\hat{y}_i - y_i\right)^2 \tag{2}$$

A.2 Adjusted R^2

Adjusted R^2, often used to assess regression models, is defined by Eq (3), where n is the sample size, k is the number of model parameters, SSE is the sum of squared errors between observations y_i (observed responses) and predictions $\hat{y}_i$ (predicted mean response) as defined in Eq (4), and SST is the sum of squared errors between observations and sample mean of observations as defined in Eq (5).

$$R^2_{\mathrm{adj}} = 1 - \frac{\frac{SSE}{n-k-1}}{\frac{SST}{n-1}} \tag{3}$$

$$SSE = \sum_{i=1}^{n}\left(y_i - \hat{y}_i\right)^2 \tag{4}$$

$$SST = \sum_{i=1}^{n}\left(y_i - \overline{y}\right)^2 \tag{5}$$

A.3 Maximum likelihood estimation

Let $X_1, \ldots, X_n$ be a random sample from a population with probability density function (pdf) $f(x|\boldsymbol{\theta})$. The likelihood function is then defined by

$$\mathcal{L}(\boldsymbol{\theta}|\mathbf{x}) = \prod_{i=1}^{n} f(x_i|\boldsymbol{\theta})$$

and the log-likelihood is similarly defined by

$$l(\boldsymbol{\theta}|\mathbf{x}) = \log \mathcal{L}(\boldsymbol{\theta}|\mathbf{x}) = \sum_{i=1}^{n} \log\left(f(x_i|\boldsymbol{\theta})\right)$$

The maximum likelihood estimator for $\boldsymbol{\theta}$ is then given by

$$\hat{\boldsymbol{\theta}} = \operatorname*{argmax}_{\boldsymbol{\theta}} \; \log \mathcal{L}(\boldsymbol{\theta}|\mathbf{x}) = \operatorname*{argmax}_{\boldsymbol{\theta}} \; l(\boldsymbol{\theta}|\mathbf{x})$$

A.4 Discrete convolutions and correlations

The discrete one-dimensional convolution of two functions $f(x)$ and $g(x)$ is defined [2] by Eq (6), and the two-dimensional convolution is defined similarly for two bivariate functions by Eq (7).

$$\left(f * g\right)(x) = \sum_{s} f(x) \cdot g(x - s) \tag{6}$$

$$\left(f * g\right)(x, y) = \sum_{s} \sum_{t} f(s, t) \cdot g(x - s, y - t) \tag{7}$$

Because the convolution operation is commutative, Eq (7) can equivalently be written as in Eq (8).

$$\left(f * g\right)(x, y) = \sum_{s} \sum_{t} f(x - s, y - t) \cdot g(s, t) \tag{8}$$

In conjunction with convolution, we also have the discrete correlation which is defined similarly as the convolution, with the aim of replacing subtraction with addition when summing over indices. Convolution implementations in programming languages usually compute the correlation; however, the convolution term usually encompasses both operations. Therefore, when we talk about a convolution between two matrices I and W, the actual operation is the correlation operation defined by Eq (9).

$$\left(I * W\right)_{x,y} = \sum_{i} \sum_{j} I_{x+i, y+j} \cdot W_{i,j} \tag{9}$$

In the context of convolutional neural networks (CNNs), the convolution is usually computed between two 3-dimensional tensors consisting of a set of feature maps and a kernel. The terms filter and kernel are used interchangeably and

denote the 3-dimensional tensor of parameters with which the feature maps are convolved. The 2-dimensional convolution between two 3-dimensional tensors $\mathcal{I} \in \mathbb{R}^{h_I \times w_I \times c}$ and $\mathcal{W} \in \mathbb{R}^{h_W \times w_W \times c}$ consists of channel-wise 2D convolutions and a summation along the third axis as shown in Eq (10).

$$(\mathcal{I} * \mathcal{W})_{x,y} = \sum_i \sum_j \sum_k \mathcal{I}_{x+i,y+j,k} \cdot \mathcal{W}_{i,j,k} \tag{10}$$

A.5 Cross-entropy and the Kullback-Leibler (KL) divergence [3]

Let X be a random variable distributed according to a pdf P, and let Q be the pdf of a proposed distribution on the same set. The entropy of $P(x)$ is defined by Eq (11), and the cross-entropy with respect to $P(x)$ is defined by Eq (12). The Kullback-Leibler divergence of $Q(x)$ with respect to $P(x)$ is defined by Eq (13).

$$H(P) = -E_{X \sim P}\left[\log P(x)\right] \tag{11}$$

$$H(P,Q) = -E_{X \sim P}\left[\log Q(x)\right] \tag{12}$$

$$D_{KL}(P||Q) = E_{X \sim P}\left[\log \frac{P(x)}{Q(x)}\right] = E_{X \sim P}\left[\log P(x) - \log Q(x)\right] \tag{13}$$

A.6 Taylor's theorem

Let $f(\mathbf{x})$ be a function that is k times differentiable at $\mathbf{a} \in \mathbb{R}^n$. Furthermore, let $\alpha = (\alpha_1, \ldots, \alpha_n)$ be defined using multi-index notation such that

$$\begin{aligned} |\alpha| &= \alpha_1 + \cdots \alpha_n \\ \alpha! &= \alpha_1! \cdots \alpha_n! \\ \mathbf{x}^\alpha &= x_1^{\alpha_1} \cdots x_n^{\alpha_n} \end{aligned}$$

and

$$\partial^\alpha = \partial_1^{\alpha_1} \cdots \partial_n^{\alpha_n}$$

Then there exists a $h : \mathbb{R}^n \to \mathbb{R}$ such that

$$f(\mathbf{x}) = \sum_{|\alpha| \leq k} \frac{\partial^\alpha f(\mathbf{a})}{\alpha!}(\mathbf{x} - \mathbf{a})^\alpha + \sum_{|\alpha| \leq k} h(\mathbf{x})(\mathbf{x} - \mathbf{a})^\alpha$$

and

$$\lim_{\mathbf{x} \to \mathbf{a}} h(\mathbf{x}) = 0$$

A.7 The logistic sigmoid function

The sigmoid function is defined by Eq (14). The function is continuous and differentiable for all $x \in \mathbb{R}$, and it is straightforward to show that its derivative is expressed by Eq (15).

$$f(x) = \frac{1}{1 + e^{-x}} \tag{14}$$

$$f'(x) = f(x) \cdot (1 - f(x)) \tag{15}$$

The derivative of the sigmoid function is always small, that is $0 < f'(x) \leq \frac{1}{4}$, and the function itself saturates to 0 or 1. The sigmoid function is commonly used in the output of artificial neural networks for binary classification problems.

B Modelling age with length

This section is concerned with the modeling age of 3540 observations of Greenland halibut (Fig 1) by using length and sex as explanatory features.

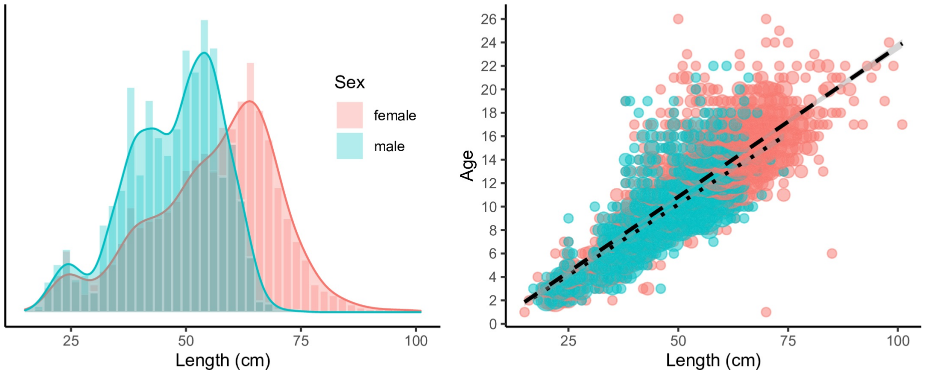

Figure 1: **Greenland halibut length distribution** *Left*: Length distribution of 3540 observations of Greenland halibut, where the female distribution has a larger mean as well as a larger variance than the male distribution. *Right*: A scatter plot of the labelled age against the measured length 3540 observations of Greenland halibut. The two dotted lines shows a fitted regression line (one for each sex).

B.1 By the von Bertalanffy growth function (VBGF)

In marine biology, the relationship between length and age is often modelled by the well known VBGF in Eq (16), where L is the length and a is the age. The parameters L_∞, k and t_0 are known as asymptotic length, growth rate, and age-at-zero, respectively.

$$L = L_\infty \left(1 - e^{-k(a-t_0)}\right) \tag{16}$$

By inverting the VBGF, we obtain Eq (17), which leads to the regression model expressed by Eq (18), where Y_i and x_i is the age and length of observation i, and z_i is an indicator variable equal to 1 if example i is male. The intercept parameter α_0 is the same for the two sexes, since it is natural to assume that males and females has equal expected length at birth.

$$a = t_0 - \frac{1}{k} \log\left(1 - \frac{L}{L_\infty}\right) \tag{17}$$

$$E(Y_i) = \alpha_0 - \alpha_1 \log\left(1 - \frac{x_i}{\beta_1}\right)(z_i - 1) - \alpha_2 \log\left(1 - \frac{x_i}{\beta_2}\right) z_i \tag{18}$$

The parameters in Eq (18) are found using numerical optimization based on the minimization of the MSE loss (section A.1) between the observed age y_i and the predicted age $\hat{y}_i$. Using *optim()* in R [6] with initial values of $\alpha_0 = 0$, $\alpha_1 = \alpha_2 = 20$ and $\beta_1 = \beta_2 = 120$, the optimal parameters were $\alpha_0 = 0.38$, $\alpha_1 = 47.23$, $\beta_1 = 251.18$, $\alpha_2 = 18.97$ and $\beta_2 = 125.03$. The MSE for the fitted values was equal to 5.80, while the adjusted R^2 (Appendix A.2) was equal to 0.67.

B.2 By linear regression

When modelling the age of Greenland halibut by length and sex using linear regression, we use the model expressed by Eq (19) where we again assume equal intersect for the two sexes. By minimising the MSE loss between the observed responses and the predictions, we obtain the parameters $\beta_0 = -1.943$, $\beta_1 = 0.256$, and $\beta_2 = -0.013$. Based on the fitted values, the MSE was equal to 5.60, while the adjusted R^2 was equal to 0.69.

$$E(Y_i) = \beta_0 + \beta_1 x_i + \beta_2 x_i z_i \tag{19}$$

The linear regression model obtained better results compared to the VBGF regression model (Eq (18)) with a loss of 5.60 compared to 5.80, and an adjusted R^2 of 0.69 compared to 0.67. We suggest therefore using Eq (19) when modelling the age of Greenland halibut using length and sex.

C Using the MSE as a loss function

Consider the regression model expressed by Eq (20) where we model the response y_i with the predictor $\mathbf{x}_i$ and the parameters $\boldsymbol{\theta}$. That is, we have a random sample $Y_i \sim (\mu_i, \sigma_i), i = 1, \ldots, n$, where ϵ_i denotes a random variable.

$$y_i = f(\mathbf{x}_i; \boldsymbol{\theta}) + \epsilon_i \tag{20}$$

C.1 Usage in regression without assumptions

If we initially assume unknown expectations and variances for the error terms in Eq (20), we can use the following derivation

$$y_i = f(\mathbf{x}_i; \boldsymbol{\theta}) + \epsilon_i \tag{21}$$
$$\epsilon_i = y_i - f(\mathbf{x}_i; \boldsymbol{\theta}) \tag{22}$$
$$(\epsilon_i)^2 = (y_i - f(\mathbf{x}_i; \boldsymbol{\theta}))^2 \tag{23}$$
$$E\left[(\epsilon_i)^2\right] = E\left[(y_i - f(\mathbf{x}_i; \boldsymbol{\theta})]^2\right] \tag{24}$$
$$\frac{1}{n}\sum_{i=1}^{n} E\left[(\epsilon_i)^2\right] = \frac{1}{n}\sum_{i=1}^{n} E\left[(y_i - f(\mathbf{x}_i; \boldsymbol{\theta})]^2\right] \tag{25}$$

where we see that the minimisation of the MSE is equivalent to minimising the sum of the expectations of the square of the error terms.

C.2 Usage in regression under the assumption of error terms with zero means and constant variance

If we assume that the expectation $E[Y_i] = \mu_i$ in Eq (20) can be modelled by a function $f(\cdot)$ such that $\mu_i = f(\mathbf{x}_i; \boldsymbol{\theta})$, we have the equivalent assumption that $E[\epsilon_i] = 0$ for all i. Then, we have the following derivation

$$y_i = f(\mathbf{x}_i; \boldsymbol{\theta}) + \epsilon_i \tag{26}$$
$$\epsilon_i = y_i - f(\mathbf{x}_i; \boldsymbol{\theta}) \tag{27}$$
$$(\epsilon_i)^2 = (y_i - f(\mathbf{x}_i; \boldsymbol{\theta}))^2 \tag{28}$$
$$E\left[(\epsilon_i)^2\right] = E\left[(y_i - f(\mathbf{x}_i; \boldsymbol{\theta})]^2\right) \tag{29}$$
$$Var(\epsilon_i) = E\left[(y_i - f(\mathbf{x}_i; \boldsymbol{\theta}))^2\right] \tag{30}$$
$$\frac{1}{n}\sum_{i=1}^{n} Var(\epsilon_i) = \frac{1}{n}\sum_{i=1}^{n} E\left[(y_i - f(\mathbf{x}_i; \boldsymbol{\theta}))^2\right] \tag{31}$$

which shows the minimization of the MSE is equivalent to minimizing the sum of the variances of the error terms. If we in addition assume constant variance for the error terms such that $\epsilon_i \sim (0, \sigma)$ for all i, we can further derive the expression

$$Var(\epsilon) = \frac{1}{n}\sum_{i=1}^{n} E\left[(y_i - f(\mathbf{x}_i; \boldsymbol{\theta}))^2\right] \tag{32}$$

which shows that the minimization of the MSE is equivalent to minimizing the variance of the error terms. This result shows that the least squares predictions are optimal in terms of expectation and variance under the assumption of $\epsilon_i \sim (0, \sigma)$.

C.3 Usage in regression under the assumption of normally distributed error terms

An additional derivation can be made if we assume that the error terms in Eq (2) are independent and identically distributed (iid) normal random variables. Then we can obtain the likelihood function of $\boldsymbol{\theta}$ (Appendix A.3) by

$$\begin{aligned}\mathcal{L}(\boldsymbol{\theta};\mathbf{y},X) &= \prod_{i=1}^{n} f(y_i;\theta,X,\sigma)\\ &= \prod_{i=1}^{n} \frac{1}{\sqrt{2\pi}\sigma} e^{-\frac{1}{2}\frac{(f(\mathbf{x}_i,\boldsymbol{\theta})-y_i)^2}{\sigma^2}}\end{aligned}$$

with the corresponding log-likelihood given by

$$l(\boldsymbol{\theta};\mathbf{y},X) = -n\cdot\log(\sqrt{2\pi}\sigma) - \frac{1}{2\sigma^2}\sum_{i=1}^{n}\left(f(\mathbf{x}_i,\boldsymbol{\theta})-y_i\right)^2$$

We find the maximum likelihood estimator for $\boldsymbol{\theta}$ by maximising the log-likelihood

$$\begin{aligned}\hat{\boldsymbol{\theta}}_{MLE} &= \underset{\boldsymbol{\theta}}{\operatorname{argmax}} \;\; -n\cdot\log(\sqrt{2\pi}\sigma) - \frac{1}{2\sigma^2}\sum_{i=1}^{n}\left(f(\mathbf{x}_i,\boldsymbol{\theta})-y_i\right)^2\\ &= \underset{\boldsymbol{\theta}}{\operatorname{argmin}} \;\sum_{i=1}^{n}\left(f(\mathbf{x}_i,\boldsymbol{\theta})-y_i\right)^2\end{aligned}$$

where the final equality denotes the MSE loss between the sample and the predictions. Thus, we see that the MSE predictions are identical to the maximum likelihood estimates for μ_i under the normal assumption.

D Xception model details

D.1 Depthwise separable convolutions

Depthwise separable convolutions were introduced as a hidden layer in CNNs under the model hypothesis that all spatial correlations and cross-channel correlations can be decoupled [1]. Depthwise separable convolutions serve as an alternative to the normal convolution (Appendix A.4) used to extract features in CNNs, and the process is a two-step procedure where an input of size $H_{in} \times W_{in} \times C_{in}$ is transformed into an output of size $H_{out} \times W_{out} \times C_{out}$. In a normal convolution, the output is obtained by convolving the input C_{out} times, using C_{out} different $m \times n$ kernels. In a depthwise separable convolution, the output is instead obtained by using the following two steps:

1. Depthwise convolution (Fig 2): Convolve each channel in the input with a $m \times n \times 1$ kernel, resulting in an intermediate output of size $H_{out} \times W_{out} \times C_{in}$.

2. Pointwise convolution (Fig 3): Convolve the intermediate output with C_{out} kernels of size $1 \times 1 \times C_{in}$, resulting in a final output of size $H_{out} \times W_{out} \times C_{out}$.

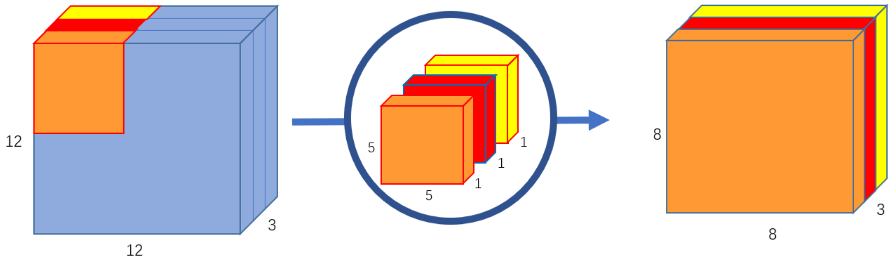

Figure 2: **Depthwise step [15]** Each input channel is convolved with a single filter before the resulting matrices are concatenated.

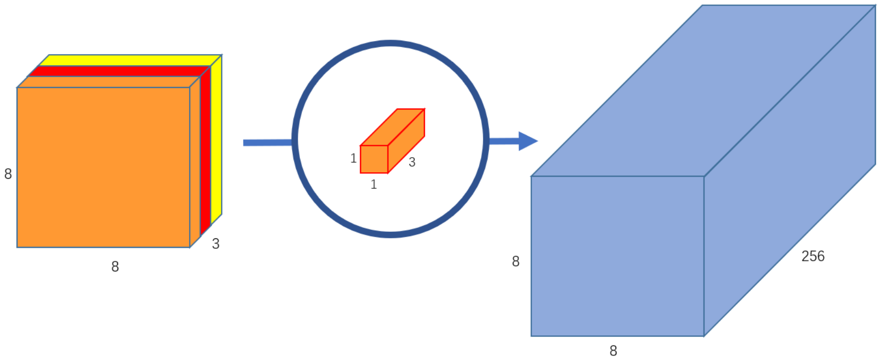

Figure 3: **Pointwise step [15]** The input is convolved with a predetermined number of 1-by-1 filters (in this case 256).

A key difference between a normal convolutional layer and a depthwise separable convolutional layer lies in the number of parameters. A convolutional layer with C_{out} units uses C_{out} kernels of size $m \times n \times C_{in}$, while a separable convolutional layer uses C_{out} kernels of size $1 \times 1 \times C_{in}$ and C_{in} kernels of size $m \times n$. Thus, the total number of parameters is $(m \cdot n \cdot C_{in} + 1) \cdot C_{out}$ for normal convolutions, versus $m \cdot n \cdot C_{in} + (C_{in} + 1) \cdot C_{out}$ parameters for separable convolutions. As an example, going from an input with 128 feature maps to an output of 256 feature maps using a filter size of 3×3, the number of parameters is reduced from approximately $300,000$ to approximately $30,000$ if convolutions are replaced by separable convolutions.

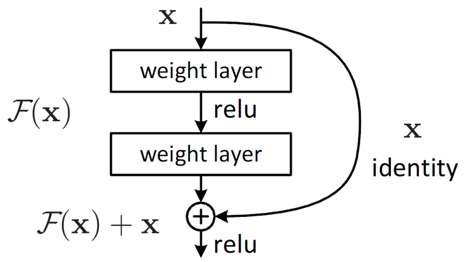

Figure 4: **Residual block** A residual block example [4] where the input bypasses two intermediate layers before being added to the input after processing.

D.2 Residual skip connections

Residual skip connections [4] provide a variation to the ordinary feedforward design of neural networks, where the concept behind residual skip connections is to add a connection between layers that are otherwise separated by intermediate layers. The idea is displayed in Fig 4, where the input to, say layer $l+1$ is given by the sum of the output from layer $l-2$ and the output from layer l. that is,

$$\mathbf{a}^{(l+1)} = f(\mathbf{a}^{(l-2)}) + \mathbf{a}^{(l-2)}$$

where $\mathbf{a}^{(l)}$ denotes the output of the layer l in the network, and where the term residual refers to the observation that the function $f(\mathbf{a}^{(l-2)})$ can be considered the residual between $\mathbf{a}^{(l+1)}$ and $\mathbf{a}^{(l-2)}$. The layers between x and the output $F(x)$ in Fig 4 are said to constitute a residual block.

Residual skip connections ensure a non-zero gradient between the output and the input of the residual block due to the addition of an identity matrix as shown in Eq (33), which ensures good convergence properties for deep neural network architectures.

$$\left(\frac{\partial \mathbf{a}^{(l+1)}}{\partial \mathbf{a}^{(l-2)}}\right) = \left(\frac{\partial \mathbf{a}^{(l)}}{\partial \mathbf{a}^{(l-1)}}\right)\left(\frac{\partial \mathbf{a}^{(l-1)}}{\partial \mathbf{a}^{(l-2)}}\right) + I \tag{33}$$

D.3 The Xception architecture

The Xception architecture [1] is an evolution of the inception models [12, 13]. All mentioned models are CNNs; however, they differ from ordinary CNNs in how the convolutions are carried out. The hypothesis of the Xception model is that all cross-channel and spatial correlations can be decoupled, and the model relies solely on separable depthwise convolutions (Appendix D.1), which replace normal convolutions for all layers except at the input. Figure 5 shows the architecture details and, although not illustrated in the figure, all convolutional operations are followed by a batch normalization layer [5]. As shown in the figure, the model uses residual connections (Appendix D.2) between blocks.

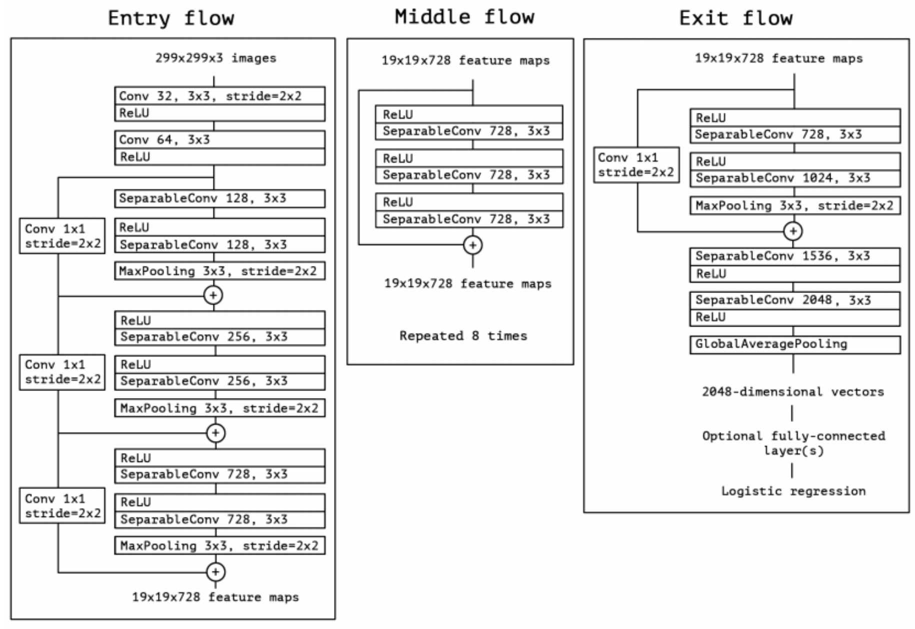

Figure 5: **Xception** Overview of the Xception architecture [1]

E Computing the KL-divergence using numerical integration

E.1 Kernel density estimation

Kernel density estimation is a non-parametric method for estimating pdf's from a sample of observations. Given a random sample $X_1, \ldots, X_n$ from a population with pdf $f(x)$, the kernel density estimator for f is given by

$$\hat{f}(x) = \frac{1}{nh} \sum_{i=1}^{n} K\left(\frac{x - X_i}{h}\right)$$

where h is a bandwidth parameter and $K(\cdot)$ is a kernel function. A range of kernel functions exists, where the Gaussian kernel function defined by the standard normal pdf

$$\phi(x) = \frac{1}{\sqrt{2\pi}} e^{-\frac{x^2}{2}}$$

is the one applied in this paper. The choice of bandwidth is not trivial, and there are a variety of bandwidth selection procedures. A comprehensive survey of methods is provided by [8]. Silverman's rule of thumb, which is defined by

$$h_{\text{SROT}} = 0.9An^{-\frac{1}{5}}$$

is used in this paper, where A is defined by

$$A = \min\left\{\hat{\sigma}, \frac{\text{IQR}}{1.34}\right\}$$

and IQR denotes the sample interquartile range.

E.2 Numerical integration of the KL-divergence

Let $\hat{f}(y)$ denote an estimated distribution of read ages obtained by kernel density estimation (Appendix E.1), and let $\hat{q}(y)$ denote the estimated distribution of ages predicted using deep learning. The KL-divergence of $\hat{q}$ with respect to $\hat{f}$ (Appendix A.5) is then defined by the formula

$$D_{KL}(\hat{f}||\hat{q}) = E_{Y\sim\hat{f}}\left[\log\frac{\hat{f}(y)}{\hat{q}(y)}\right] = \int_{-\infty}^{\infty}\hat{f}(y)\log\left(\frac{\hat{f}(y)}{\hat{q}(y)}\right)dy \tag{34}$$

This expression is intractable to compute analytically, and we instead compute the integral numerically using a selection of points from $\hat{f}$ and $\hat{q}$. The integrals in this paper are computed using Simpson's rule [7].

F Interpretability techniques

This section provides a short description of the feature relevance techniques applied to the deep learning model used in this paper.

F.1 Gradient saliency maps

Gradient saliency maps [9] are based on a simple approach to attributing feature relevance to the input. Using Taylor's theorem (Appendix A.6), the first-order approximation of a function $f(\mathbf{x})$ around a point $\mathbf{x}_0$ is given by Eq (35).

$$f(\mathbf{x}) = f(\mathbf{x}_0) + \left(\nabla_{\mathbf{x}} f(\mathbf{x})|_{\mathbf{x}=\mathbf{x}_0}\right)^T(\mathbf{x}-\mathbf{x}_0) \tag{35}$$

In the context of neural networks, where the function $f(\cdot)$ denotes the output of the model and $\mathbf{x}_0$ denotes a vector of all the input features for the input in question, the neural network can be approximated by the linear function

$$f(\mathbf{x}) = \mathbf{w}^T\mathbf{x} + b$$

where the weights of the linear function are given by the gradient

$$\mathbf{w} = \nabla_{\mathbf{x}} f(\mathbf{x})|_{\mathbf{x}=\mathbf{x}_0}$$

and the bias is given by the constant

$$b = f(\mathbf{x}_0) - \left(\nabla_{\mathbf{x}} f(\mathbf{x})|_{\mathbf{x}=\mathbf{x}_0}\right)^T\mathbf{x}_0$$

Since the gradients constitute the weights of the function, the gradients in this sense represent the relative importance of each feature. That is, the features with derivatives of the largest magnitudes are the pixels that would change the output the most by being modified the least. In this approach, the relevance attribution for the input feature i is given by

$$R_i = \left.\frac{\partial f(\mathbf{x})}{\partial x_i}\right|_{\mathbf{x}=\mathbf{x}_0}$$

F.2 Baseline gradients: a gradient approach using baseline inputs

The gradient-based method in Appendix F.1 is based on an argument involving Taylor's theorem. Another application of Taylor's Theorem can be applied as follows. Let $f(\cdot)$ be the function defined by our neural network, and let $\mathcal{X}$ and $\mathcal{X}_0$ be input and baseline, respectively, with $\mathcal{H} = \mathcal{X} - \mathcal{X}_0$. Using a first-order Taylor approximation (Appendix A.6), we can write $f(\mathbf{x})$ as

$$\begin{aligned} f(\mathbf{x} - \mathbf{h}) &= f(\mathbf{x}) + (\nabla_{\mathbf{x}} f)^T \mathbf{h} \\ f(\mathbf{x}_0) &= f(\mathbf{x}) + (\nabla_{\mathbf{x}} f)^T (\mathbf{x} - \mathbf{x}_0) \\ &\Rightarrow \\ f(\mathbf{x}) - f(\mathbf{x}_0) &= (\nabla_{\mathbf{x}} f)^T (\mathbf{x} - \mathbf{x}_0) \end{aligned}$$

resulting in the relevance attribution rule

$$R_i = \frac{\partial f(\mathbf{x})}{\partial x_i} \cdot (x_i - x_{0,i})$$

This is nearly identical to the gradient saliency maps described in section F.1. The important difference is that we are estimating the feature-wise contribution to the output difference, where the difference is measured with respect to the baseline. In situations where a baseline of zeros is appropriate, this simplifies the calculation

$$f(\mathbf{x}) - f(\mathbf{x}_0) = (\nabla_{\mathbf{x}} f)^T \mathbf{x}$$

thus, we can guarantee that only non-zero features are given non-zero scores.

F.3 Guided Backpropagation

Guided backpropagation was introduced in conjunction with a fully convolutional network using ReLU activations [10], as a feature attribution method that is identical to the procedure to compute gradients, except for ReLU activations. In explaining the method, we first note that the gradient of the output $a^{(L)}$ from the layer L with respect to an input $\mathbf{x}$ is given by

$$\nabla_{\mathbf{x}} a^{(L)} = \left(\left(\frac{\partial \mathbf{a}^{(1)}}{\partial \mathbf{x}} \right) \left(\frac{\partial \mathbf{a}^{(2)}}{\partial \mathbf{a}^{(1)}} \right) \cdots \left(\frac{\partial \mathbf{a}^{(L-1)}}{\partial \mathbf{a}^{(L-2)}} \right) \right)^T \nabla_{\mathbf{a}^{(L-1)}} a^{(L)}$$

We also note that when using the ReLU activation function, each element in a matrix $\left(\frac{\partial \mathbf{a}^{(l+1)}}{\partial \mathbf{a}^{(l)}}\right)$ can be written as

$$\frac{\partial a_j^{(l+1)}}{\partial a_k^{(l)}} = w_{jk}^{(l+1)} \cdot f'(z_j^{(l+1)}) = \begin{cases} w_{jk}^{(l+1)}, & z_j^{(l+1)} > 0 \\ 0, & \text{otherwise} \end{cases}$$

Thus, ReLU activations ensure that only gradients corresponding to positive activations are propagated backward. Note, however, that this does not require that the weights be positive, but only that the sum

$$z_j^{(l+1)} = \sum_{k=1}^{n} w_{jk}^{(l+1)} a_k^{(l)} + b_j^{(l+1)}$$

is positive. Thus, it is still possible for activations contributing negative to the score to get attributed by backpropagation. In guided backpropagation however, this is avoided by the additional requirement

$$\frac{\partial a_j^{(L)}}{\partial a_k^{(l+1)}} > 0$$

resulting in the relevance attribution rule

$$R_j^{(l)} = \sum_k R_{jk}^{*(l+1)}$$

where

$$R_{jk}^{*(l+1)} = \begin{cases} w_{jk}^{(l+1)} \cdot R_k^{(l+1)}, & z_j^{(l+1)} > 0, \;\; R_k^{(l+1)} > 0 \\ 0, & \text{otherwise} \end{cases}$$

Thus, for a network with rectified linear units, guided backpropagation will give a zero attribution to any activation that at some point contributes negatively to the output score.

F.4 Integrated Gradients

Integrated gradients [11] is a proposed feature relevance attribution method that is based on an axiomatic approach to determining input feature attributions. The idea is to compare the input of interest with a baseline input and to determine the feature-wise contribution to the difference in model output for the two inputs. The authors propose that relevance attribution methods should comply with the following axioms.

- Sensitivity: A feature should be attributed if a change in that feature for the input results in a change in the output. If the network output doesn't depend on that feature, the attribution for that feature should be zero.
- Invariance: A feature relevance attribution method applied to different models with identical outputs for all identical inputs should produce identical attributions for all models.
- Completeness: The sum of all feature attributions should equal the difference between the output of the model at the input, minus the output for the model at the baseline.
- Linearity: The attributions should preserve any linearity within the network. That is, if the output is a weighted sum of two models, the attributions to the input should be a weighted sum of the attributions with respect to the two models.

Integrated gradients is described by the following. Let $\mathbf{x} \in \mathbb{R}^n$ be an input vector, $\mathbf{x}' \in \mathbb{R}^n$ be a baseline vector, and $f(\mathbf{x})$ be the output of a neural network for input $\mathbf{x}$. The integrated gradients of $\mathbf{x}$ is defined as

$$\text{IntegratedGradients}_i(\mathbf{x}) \equiv (x_i - x_i') \cdot \int_{\alpha=0}^{1} \left. \frac{\partial f(\mathbf{y})}{\partial y_i} \right|_{\mathbf{y}=\mathbf{x}'+\alpha(\mathbf{x}-\mathbf{x}')} d\alpha \tag{36}$$

It is shown [11] that integrated gradients indeed satisfy the proposed axioms and that a fundamental theoretical property of integrated gradients is the fact that the sum of all integrated gradients equals the score for the input, minus the score for the baseline, that is,

$$\sum_{i=1}^{n} \text{IntegratedGradients}_i(\mathbf{x}) = f(\mathbf{x}) - f(\mathbf{x}')$$

Note that the integral in Eq (36) is in practice computed using numerical integration. The fact that integrated gradients satisfy the completeness axiom can be used as a mean to check computations and measure the accuracy of the numerical integration.

F.5 Integrated guided gradients

The last example did not include guided backpropagation (F.3), however since guided backpropagation is a modification of gradient saliency maps, similar problems as those demonstrated for gradient based methods will be present in the guided backpropagation case as well. Therefore, an alternative to guided backpropagation is provided by combining integrated gradients with guided backpropagation. If we let $R(x_i)$ denote the relevance attribution for feature i, obtained by guided backpropagation, then the integrated guided gradient of feature i is given by

$$\text{IntegratedGuidedGradients}_i(\mathbf{x}) = (x_i - x_i') \cdot \int_0^1 R(x_i' + \alpha(x_i - x_i'))d\alpha$$

This method attempts to combine the sparse attributions given by guided backpropagation with the good theoretical properties of integrated gradients.

F.6 A simple comparison of feature attribution methods

Consider a function defined by

$$f(x_1, x_2) = \sigma(x_1) + x_2 \tag{37}$$

where σ denotes the logistic sigmoid function described in Appendix A.7 which has the property

$$\frac{\partial \sigma(x)}{\partial x} = \sigma(x)(1 - \sigma(x))$$

The feature relevance attribution provided by the gradient of f (Appendix F.1) is thus given by

$$\text{Gradients}(\mathbf{x}) = \begin{bmatrix} \sigma(x_1)\,(1 - \sigma(x_1)) \\ 1 \end{bmatrix}$$

and the baseline gradients (Appendix F.2) are given by

$$\text{BaselineGradients}(\mathbf{x}) = \begin{bmatrix} \sigma(x_1)\,(1 - \sigma(x_1)) \cdot (x_1 - x_1') \\ (x_2 - x_2') \end{bmatrix}$$

The integrated gradient is a little less straight forward to compute, but we have for the first feature that

$$\begin{aligned} \text{IntegratedGradients}(x_1) &= (x_1 - x_1') \cdot \int_0^1 \sigma(x_1' + \alpha(x_1 - x_1'))(1 - \sigma(x_1' + \alpha(x_1 - x_1')))d\alpha \\ &= (x_1 - x_1') \cdot \frac{1}{x_1 - x_1'} \cdot \sigma(x_1' + \alpha(x_1 - x_1'))\Big|_0^1 \\ &= \sigma(x_1) - \sigma(x_1') \end{aligned}$$

whereas the integrated gradient for the second feature is given by

$$\begin{aligned} \text{IntegratedGradients}(x_2) &= (x_2 - x_2') \cdot \int_0^1 d\alpha \\ &= x_2 - x_2' \end{aligned}$$

To summarise, we have the following relevance attributions for the three methods.

$$
\begin{aligned}
\text{Gradients}(\mathbf{x}) &= \begin{bmatrix} \sigma(x_1)\,(1-\sigma(x_1)) \\ 1 \end{bmatrix} \\
\text{BaselineGradients}(\mathbf{x}) &= \begin{bmatrix} \sigma(x_1)\,(1-\sigma(x_1)) \cdot (x_1 - x'_1) \\ (x_2 - x'_2) \end{bmatrix} \\
\text{IntegratedGradients}(\mathbf{x}) &= \begin{bmatrix} \sigma(x_1) - \sigma(x'_1) \\ (x_2 - x'_2) \end{bmatrix}
\end{aligned}
$$

Now consider an input where x_1 is large and x_2 is zero, that is, $x_1 \to \infty$, such that the first term in Eq (37) will converge to 1, while the second term will be 0. However, the feature relevance attributions will differ for the three methods. If we assume that we are using a baseline input of $(0, 0)$ and using the fact that

$$
\lim_{x\to\infty} \sigma(x)(1-\sigma(x)) = 0
$$

and

$$
\lim_{x\to\infty} \sigma(x)\,(1-\sigma(x)) \cdot x = 0
$$

the resulting feature relevance attributions are

$$
\begin{aligned}
\text{Gradients}(\mathbf{x}) &\to \begin{bmatrix} 0 \\ 1 \end{bmatrix} \\
\text{BaselineGradients}(\mathbf{x}) &\to \begin{bmatrix} 0 \\ 0 \end{bmatrix} \\
\text{IntegratedGradients}(\mathbf{x}) &\to \begin{bmatrix} 1 \\ 0 \end{bmatrix}
\end{aligned}
$$

which displays three completely different results. We see that the gradient-based methods fail to capture what the integrated gradients does; namely that only the first feature contributes to the function output. This example suggests an inherent weakness in gradient saliency maps and it is argued [11] that integrated gradients possess better theoretical properties.

References

[1] François Chollet. *Xception: Deep Learning with Depthwise Separable Convolutions.* 2017. arXiv: `1610.02357 [cs.CV]`.

[2] Rafael C. Gonzalez and Richard E. Woods. *Digital image processing.* Upper Saddle River, N.J.: Prentice Hall, 2008. URL: `http://www.amazon.com/Digital-Image-Processing-3rd-Edition/dp/013168728X`.

[3] Ian Goodfellow, Yoshua Bengio, and Aaron Courville. *Deep Learning.* `http://www.deeplearningbook.org`. MIT Press, 2016.

[4] Kaiming He et al. *Deep Residual Learning for Image Recognition.* 2015. arXiv: 1512.03385 [cs.CV].

[5] Sergey Ioffe and Christian Szegedy. “Batch normalization: Accelerating deep network training by reducing internal covariate shift”. In: *CoRR, abs/1502.03167* (2015). URL: http://arxiv.org/abs/1502.03167.

[6] RStudio Team. *RStudio: Integrated Development Environment for R.* RStudio, Inc. Boston, MA, 2019. URL: http://www.rstudio.com/.

[7] Timothy Sauer. *Numerical Analysis.* 2nd. USA: Addison-Wesley Publishing Company, 2011. ISBN: 0321783670.

[8] Simon J. Sheather. “Density Estimation”. In: *Statistical Science* 19.4 (2004), pp. 588–597. DOI: 10.1214/088342304000000297. URL: https://doi.org/10.1214/088342304000000297.

[9] Karen Simonyan, Andrea Vedaldi, and Andrew Zisserman. *Deep Inside Convolutional Networks: Visualising Image Classification Models and Saliency Maps.* 2014. arXiv: 1312.6034 [cs.CV].

[10] Jost Tobias Springenberg et al. *Striving for Simplicity: The All Convolutional Net.* 2015. arXiv: 1412.6806 [cs.LG].

[11] Mukund Sundararajan, Ankur Taly, and Qiqi Yan. *Axiomatic Attribution for Deep Networks.* 2017. arXiv: 1703.01365 [cs.LG].

[12] Christian Szegedy et al. *Going Deeper with Convolutions.* 2014. arXiv: 1409.4842 [cs.CV].

[13] Christian Szegedy et al. *Rethinking the Inception Architecture for Computer Vision.* 2015. arXiv: 1512.00567 [cs.CV].

[14] Sergios Theodoridis and Konstantinos Koutroumbas. *Pattern Recognition, Fourth Edition.* Academic Press, 2009. ISBN: 9781597492720.

[15] Chi-Feng Wang. *A Basic Introduction to Separable Convolutions.* 2018. URL: https://towardsdatascience.com/a-basic-introduction-to-separable-convolutions-b99ec3102728.
